# The Multidisciplinary Guidelines for Diagnosis and Referral in Cerebral Visual Impairment

**DOI:** 10.3389/fnhum.2022.727565

**Published:** 2022-06-30

**Authors:** Frouke N. Boonstra, Daniëlle G. M. Bosch, Christiaan J. A. Geldof, Catharina Stellingwerf, Giorgio Porro

**Affiliations:** ^1^Royal Dutch Visio, National Foundation for the Visually Impaired and Blind, Huizen, Netherlands; ^2^Department of Cognitive Neuroscience, Donders Institute for Brain, Cognition and Behavior, Radboud University Medical Centre Nijmegen, Nijmegen, Netherlands; ^3^Behavioral Science Institute, Radboud University, Nijmegen, Netherlands; ^4^Department of Clinical Genetics, Erasmus MC, Rotterdam, Netherlands; ^5^Department of Ophthalmology, UMC Utrecht and Amphia Hospital Breda, Breda, Netherlands

**Keywords:** cerebral visual impairment, visual development, visually impaired children, perinatal damage, congenital anomaly of brain, visual behavior analysis, visual functions, MRI in VI children

## Abstract

**Introduction:**

Cerebral visual impairment (CVI) is an important cause of visual impairment in western countries. Perinatal hypoxic-ischemic damage is the most frequent cause of CVI but CVI can also be the result of a genetic disorder. The majority of children with CVI have cerebral palsy and/or developmental delay. Early diagnosis is crucial; however, there is a need for consensus on evidence based diagnostic tools and referral criteria. The aim of this study is to develop guidelines for diagnosis and referral in CVI according to the grade method.

**Patients and Methods:**

We developed the guidelines according to the GRADE method 5 searches on CVI (children, developmental age ≤ 18 years) were performed in the databases Medline, Embase, and Psychinfo, each with a distinct topic.

**Results:**

Based on evidence articles were selected on five topics: 1. Medical history and CVI-questionnaires 23 (out of 1,007). 2. Ophthalmological and orthoptic assessment 37 (out of 816). 3. Neuropsychological assessment 5 (out of 716). 4. Neuroradiological evaluation and magnetic resonance imaging (MRI) 9 (out of 723). 5. Genetic assessment 5 (out of 458).

**Conclusion:**

In medical history taking, prematurity low birth weight and APGAR (Appearance, Pulse, Grimace, Activity, Respiration) Scores (<5) are important. Different questionnaires are advised for children under the age of 3 years, older children and for specific risk groups (extremely preterm). In ophthalmological examination, eye movements, specially saccades, accommodation, crowding, contrast sensitivity and visual fields should be evaluated. OCT can show objective signs of *trans*-synaptic degeneration and abnormalities in fixation and saccades can be measured with eye tracking. Screening of visual perceptive functioning is recommended and can be directive for further assessment. MRI findings in CVI in Cerebral Palsy can be structured in five groups: Brain maldevelopment, white and gray matter lesions, postnatal lesions and a normal MRI. In children with CVI and periventricular leukomalacia, brain lesion severity correlates with visual function impairment. A differentiation can be made between cortical and subcortical damage and related visual function impairment. Additional assessments (neurological or genetic) can be necessary to complete the diagnosis of CVI and/or to reveal the etiology.

## Introduction

Cerebral visual impairment (CVI) has been defined as a verifiable visual dysfunction which cannot be attributed to disorders of the anterior visual pathways or any potentially co-occurring ocular impairment (Sakki et al., 2018). CVI is caused by abnormalities in the retro-geniculate system and not by pathology of the eye, however, CVI can occur together with pathology of the eye for instance in retinopathy of prematurity (ROP). CVI greatly affects development in all its aspects (Boonstra et al., 2012; Ego et al., 2015) and therefore, diagnosing CVI in early life is essential to enable early and tailor made interventions (Fazzi et al., 2009). Because there is a need for clear guidelines, to structure the diagnostic procedure in this heterogeneous group, we describe the guidelines for diagnosis and referral of CVI based on five literature searches.

Cerebral visual impairment is the most common cause of visual impairment in children in western countries. Perinatal hypoxic-ischemic damage is the most frequent cause of CVI, but the etiology is variable. Hypoxic Ischemic Injury (HII) is the most common cause of CVI 60% of the children with HII also has CVI (Cioni et al., 1996; Good et al., 2001; Fazzi et al., 2007; Solebo et al., 2017; Sakki et al., 2018). Another important cause of CVI is developmental anomaly of brain (genetic).

Problems with processing of visual information and or integration of visual information are symptoms of higher order visual functions (Good et al., 2001; Fazzi et al., 2007; Philip and Dutton, 2014). In children with these impairments, a relatively good visual acuity can be found.

In ophthalmological examination associated ocular and oculomotor deficits can be found such as strabismus, nystagmus, motility disorders of the eye, delay in saccades, pallor of the optic nerve and visual field abnormalities. In CVI retro-geniculate damage results in ganglion cell layer thinning due to retrograde *trans*-synaptic degeneration (RTSD) (Jacobson and Dutton, 2000; Hoyt, 2003; Khetpal and Donahue, 2007; Bosch et al., 2014b; Jacobson et al., 2019). In CVI these ocular and oculomotor deficits are often found in both eyes and sometimes show the same outcome in right and left eye. In addition, a clear clinical picture can be found as a result of damage or congenital abnormalities in different stages of the development of the visual system:

• Congenital abnormalities of brain and deviations in brain development.
• Perinatal damage.
• Postnatal damage.

Children with CVI are not always visually impaired, according to the WHO definition (visual acuity < 6/18). Children with CVI may have a better visual acuity (mildly impaired 6/12 to 6/18 or even better). CVI is often caused by HIE (Hypoxic Ischemic Encephalopathy), which is found in 6/1,000 life born infants in western countries and about 26/1,000 in non-western countries. 60% of these children have CVI (Fazzi et al., 2007; Solebo et al., 2017).

Another important cause of CVI is congenital anomaly of brain as a result of a genetic anomaly. Children with congenital anomalies of brain are not always referred to an ophthalmologist, therefore the incidence of CVI might be even higher. Children with a history of perinatal damage or congenital anomaly of brain need examinations specific for CVI because they often are not aware of their limitations and a majority has no specific ophthalmological complaints (Sakki et al., 2021). However, they can have slow eye movements (saccades), crowding (the impairment of the ability to recognize objects in clutter), and impairment of accommodation or complaints that are related to impairment of processing of visual information (Bosch et al., 2014b). Children with CVI can also show several peculiar behavioral signs; short visual attention span; markedly fluctuating visual performances; the need for time, environmental stability, and repetition of items to obtain the best response (Fazzi et al., 2007).

Demarcation of content: In the developing process of the guidelines a clear definition and description of the patient group was necessary. The group in this study contains children between 0 and 18 years with CVI caused by various diseases, manifesting with various complaints and function deficits. Often co-morbidity is found. After the decision to study five topics the authors have chosen for a practical approach; firstly, articles were selected where the abbreviation CVI was used. Articles about impairment of visual functions may therefore not always have been included and the abbreviation CVI may have been defined differently in different articles. Five topics have been selected to focus upon, following a bottleneck analysis by the involved national professional associations: The Dutch ophthalmological organization, the organization of clinical geneticist, neonatologists, neurologists, radiologists, rehabilitation physicians, physicians for multiple impaired and youth healthcare physicians, the society for child and hospital and the society for patients with visual impairment.

The five topics are also mentioned by McConnell et al. (2021) who described the assessments currently used to investigate and diagnose CVI. According to Mc Connell, there is a lack of common practice in the approaches used by clinicians to investigate and diagnose CVI in children. At present, a “diagnosis of exclusion” remains the most common means to diagnose CVI. Therefore development of clinical guidelines for assessment and diagnosis are necessary to ensure consistency in the diagnosis of CVI and the timely implementation of support to alleviate the impact of CVI on the child’s daily living. The aim of this study is to develop guidelines for diagnosis and referral in CVI according to the grade method.

The topics:

1 Medical history and CVI-questionnaires.
2 Ophthalmological and orthoptic assessments in CVI.
3 Neuropsychological assessment.
4 Neuroradiological evaluation and MRI.
5 Genetics assessment.

### Medical History and Cerebral Visual Impairment-Questionnaires

In general, children with CVI enter the diagnostic circuit in three ways:

1 Parents worry about the visual functioning of their child.
2 Children with an increased risk on CVI, without a specific question of the parents. In this group, active screening is necessary using a CVI questionnaire.
3 Children with an intellectual disability or syndrome who can be screened on visual functioning.

### Ophthalmological and Orthoptic Assessments in Cerebral Visual Impairment

If children with possible visual impairment or deviations of visual behavior, are referred to an ophthalmologist a series of test possibilities are available. We aimed to identify which tests are relevant for the diagnosis CVI.

### Neuropsychological Assessment

If the history or the ophthalmological and orthoptic or psychological examination shows indications for disorders in visual information processing and when ophthalmological findings provide insufficient explanation neuropsychological assessment can be useful to aid the diagnosis.

Although it is recommended to perform a neuropsychological assessment in children with possible CVI (Fazzi et al., 2009; Boot et al., 2010; Ortibus E. L. et al., 2011), there is no consensus in terms of a diagnostic protocol. Therefore, professionals have no reference for the appropriate age, domains of functioning and tests to include in the assessment.

### Neuroradiological Evaluation and Magnetic Resonance Imaging

Imaging is part of the diagnostic possibilities in CVI. Neuroradiological imaging in children with CVI could be applied to make an inventory of the damage of those parts of the brain that are concerned in visual processing.

In this question we can define two sub questions:

1 What is the prognostic value of MRI in children with a risk on CVI?
2 In children with deviant viewing behavior that have been referred because of suspected CVI, what is the role of MRI in the differential diagnosis of CVI.

Several anomalies of brain can be found in CVI. Such as embryological deviations (Encephalocele, holoprosencephalies) cortical developmental malformations (occipital lissencephaly, pachygyria, polymicrogyria, and schizencephaly) periventricular leukomalacia (PVL), periventricular and intraventricular hemorrhages, neonatal encephalopathy, or cerebral ischemia after the perinatal period (Hoyt and Taylor, 2017).

These congenital malformations of the brain can result in damage to the visual pathways. In preterm born babies a lack of oxygen results in white matter damage, while full term born babies are at risk for gray matter damage (cortex). Cerebral damage can result in a large range of CVIs varying from hardly noticeable visual field impairment to severe visual field impairment and even blindness. Individuals with CVI exhibit a wide range of visual deficits and, in particular, present with impairments of higher order visual spatial processing (referred to as “dorsal stream dysfunction”) as well as object recognition (associated with processing along the ventral stream; Bennett et al., 2020). The value of neuroradiological imaging is not yet clearly defined in this group of patients. Guidance is warranted for the application of neuroradiological examination in this population and its role in the diagnosis CVI. Because of the radiation burden from CT-scans in childhood (Pearce et al., 2012) and the fact that ultrasound is not possible after the age of 1 year this chapter focusses on MRI (McGuirt, 2016).

### Genetic Assessment

Traditionally, CVI was seen as a result of perinatal damage. Nowadays, it is clear that CVI can also be caused by several genetic disorders (Bosch et al., 2014b,a; Hoyt and Taylor, 2017). This recent knowledge, however, is not yet well known by professionals. Therefore, CVI is often still defined as a result of perinatal damage and children with CVI as a result of a genetic disorder might not receive the correct visual rehabilitation interventions. This search question aimed to investigate in which genetic disorders an investigation for CVI is warranted.

## General Methods (All Topics)

To formulate the guidelines according to the GRADE method, searches for five topics on CVI (children, developmental age ≤ 18 years) were performed in the databases Medline, Embase and Psych info.

### AGREE and GRADE

The guidelines were formulated according to the AGREE II method (Appraisal of Guidelines for Research & Evaluation II; Brouwers et al., 2010). This is a well-structured method which commences with a bottleneck analysis: With the different disciplines involved priority was given to specific topics based on medical and organizational arguments. This was followed by a discussion on the concepts of search questions. Together this emerges into final search questions. The searches were performed for systematic reviews, SIGN, Medline, and with specific key words for each search question in Medline, Embase, Psych info. The guideline research group selected relevant articles, based on the pre-defined selection criteria. The articles selected were used to answer the search questions for each search topic.

For quality criteria of the different studies, we used instruments to reduce the risk of bias as recommended by the Cochrane Collaboration: AMSTAR for systematic reviews, Cochrane for randomized controlled research trials, ACROBAT-NRS for observational research; QUADAS II, for diagnostic Research. The relevant results of the articles were described in evidence tables (Supplementary Appendix). The most important results from literature were summarized in the literature summary of each search. If sufficient studies were available, a quantitative summary (meta-analysis) was given. For the rating of diagnostic tests or etiology or prognostic value, the GRADE ranking was used (Grading Recommendations Assessment, Development and Evaluation^1^). In GRADE, four levels of evidence of scientific prove are described: High, Moderate, Low and very low, with the starting aim at high with downgrading for the risk of bias, inconsistency, indirectness, imprecision, and publication bias.

Levels of evidence referring to the amount of evidence based on the outcome of literature (Guyatt et al., 2008) are:

• Very low: the true effect is probably markedly different from the estimated effect.
• Low; the true effect might be markedly different from the estimated effect.
• Moderate; the authors believe that the true effect is probably close to the estimated effect.
• High; the authors have a lot of confidence that the true effect is similar to the estimated effect.

The outcome of the searches was assessed by two authors for each search, in a predefined procedure; Each author selected relevant articles if there was a large difference in selection, the articles were selected with the help of a third independent person.

### PICO

In order to define the systematic search, the characteristics of the research questions were defined in a PICO.

Definitions were used as mentioned in the different articles. PICO stands for:

P: Patient group: The specific target group that the research question is about.
I: Intervention: The intervention or test or assessment that the research question is about.
C: Comparison: Alternative to Intervention which is applicable to the research question.
O: Outcome: The desired intervention, assessment or test for the research question.

### Search, General Inclusion Criteria (All Topics)

The searches were performed in March-July 2017 and in December 2021 for publications dealing with etiology, risk factors and clinical history elements of CVI in Medline (OVID) and Embase^2^ using relevant terms and in the topic on neuropsychological assessment PsychInfo was used because of the content. The searches have been carried out by a clinical librarian of the Dutch Institute for Medical Specialists and the clinical librarian of the Radboud Medical center. Inclusion criteria were:

- Systematic reviews.
- Randomized controlled trials.
- Cohort studies and case series with more than five patients, no case reports were included.
- Written in English.
- Age of the children ranging from 0 to 18 years developmental age.
- Diagnosed with CVI as defined in the introduction.
- Start of outcome population according to the definition of CVI in the introduction and published in peer reviewed Journals.
- Population start and end outcome: according to the CVI definition in the introduction. Studies with an unclear metabolic disorder or without DNA diagnosis were excluded. CVI was due to *etiological* conditions occurring between ages 2 and 12 years, because all causes of damage during development of the visual system can cause CVI. Articles dealing with acquired brain injury which was caused after the age of 12 years, which means after major development of the visual system, were *excluded* as well as those describing functional visual disturbance. Acquired brain injury studies with etiology between 3 and 12 years (during the major development of the visual system) and published in peer-reviewed journals were *included*. Studies on learning and behavioral problems were excluded in all topics but topic 3 (neuropsychological assessment).

In this study the excluded literature is not mentioned in the references. The exclusion tables for this study can be requested.

### Topics

Based on evidence, articles were selected on the five topics mentioned in the introduction a control or comparison was mentioned if applicable.

The search outcome of each topic was screened and selected by two authors. In case of conflicting outcome a third independent person, with knowledge of the topic, was involved to decide on the selection.

#### Topic One: Medical History and Questionnaires

Questions:

1A How should screening on CVI be performed?
1B Which questionnaires should be used in case of suspected CVI? In addition, for which CVI subgroup should the questionnaires be applied?

PICO:

For question 1A: Which elements should be included in the clinical history to screen for CVI?

P: Patients: Children who possibly have CVI.
I: Investigation: Medical and perinatal history, congenital brain anomalies, developmental delay, and genetic diagnosis.
C: Comparison (control): Children with learning problems or behavioral problems and characteristics of delayed visual development.
O: Outcome: Sensitivity and specificity of medical history questions for the diagnosis of CVI.

Outcome measures were not defined *a priori*; the definitions mentioned in the studies were used.

For question 1B: Which questionnaire should be used in case the professional has a suspicion of CVI, based on the clinical history?

P: Patients with unusual visual behavior, problems with visual orientation, fixation problems, slow vision (delayed reactions on visual stimuli), predominance of sound over vision, problems with visual selection, crowding difficulties, visual field preference, visual attention, and object recognition.
I: Investigation: CVI screening questionnaires.
C: Comparison: -
O: Outcome: Sensitivity/Specificity, clinometric aspects.

Clinometric properties such as sensitivity, specificity and diagnostic value were deemed critical outcome measures.

Search and selection:

A search was performed for publications dealing with etiology, risk factors and clinical history elements of CVI in Medline (OVID) and Embase (see text footnote 2) using relevant terms. The search string is shown below (Supplementary Appendix Figure A).

Studies were selected based on the criteria mentioned in general methods.

The search resulted in 1,007 articles. Articles dealing with acquired brain injury after the age of 12 years were excluded as well as those describing functional visual disturbance.

Based on title and abstract review, 75 articles were selected. Of those, for Question 1A, 14 articles were withheld (Supplementary Appendix Table 1A). For Question 1B 9 studies were included for analysis (Supplementary Appendix Table 1B).

To prepare the search on clinical history and questionnaires, we first classified groups at risk for CVI by obtaining the clinical history, because based on this selection, caretakers and professionals can be alert on the risk of CVI. Secondly, the use of questionnaires is studied. Are there questionnaires that can be used as an instrument to screen on CVI? For which groups are these questionnaires applicable? Based on the outcome of a questionnaire the children could be referred selectively for an ophthalmologic and neuro-radiological and/or neuropsychological assessment.

#### Topic Two: Ophthalmological and Orthoptic Assessment

Questions:

Which are the ophthalmological and orthoptic assessments that are required in case of suspected CVI in a general hospital?
Which ophthalmological and orthoptic assessments can be performed in academic centers in case of suspected CVI?

The question is focused on ophthalmologists and orthoptists because these two groups of professionals often work together in case of complex neuroophthalmological pathology.

PICO:

In order to define the systematic search, the characteristics were defined in a PICO.

P: Patient: patients with unusual visual behavior who have been referred to the ophthalmologist.
I: Investigation: Ophthalmological and orthoptic investigation.
C: Comparison: -
O: Outcome: Specificity and sensitivity of tests in CVI.

Definitions were used as mentioned in the different articles.

Search and selection:

In Medline, a search was performed in April 2017 and December 2021, using relevant search terms to select studies about ophthalmological and orthoptic assessment in a peripheral hospital setting. Selected were 816 articles. Studies were selected based on the criteria mentioned in general methods.

Question 2A and B: Based on title and abstract at first 83 studies were selected of which 27 studies were excluded (other topic or small group) and after full text reading 19 studies were excluded and 37 studies remained included. Outcomes of these (A:16; B:21) searches have been included in the literature analysis. The most important study characteristics and results are represented in the evidence table (Supplementary Appendix Tables 2A, 2B).

#### Topic Three: Neuropsychological Assessment

In order to apply neuropsychological assessment in children in CVI appropriately and in a functional way it has to be clear:

1 Which aspects of visual attention and visual perceptive functions have to be examined?
2 Which neuropsychological tests are available for this assessment?
3 Which test outcome is relevant for the diagnosis CVI?
4 When abnormal test results do not support a CVI-diagnosis (i.e., differential diagnosis)?

Transparency about the content of a neuropsychological examination can add to an efficacious diagnostic procedure in CVI and appropriate and timely referral and rehabilitation.

Question 3: Which neuropsychological tests can be used in the neuropsychological assessment of children with CVI?

To define the search question a systematic literature analysis is performed for the following question.

P: Patients: Children suspected of having CVI.
I: Investigation: Neuropsychological test.
C: Comparison (control): No tests or other tests.
O: Outcome: Diagnostic value and accuracy.

Search and selection:

In the databases Medline (OVID), Psych INFO (OVID) were searched in June 2017 and December 2021 using relevant search terms for neuropsychological assessment in the context of CVI diagnostics. For this search PsychInfo was used because of the content (neuropsychological tests). The search resulted in 716 hits. The search string is shown below (Supplementary Appendix Figure C). Studies were selected based on the criteria mentioned in general methods. Based on title and abstract 29 studies were initially selected (Supplementary Appendix Table 3). After full text reading 24 studies were excluded and 5 studies were included. Studies on acquired brain injury in older children and studies on functional visual impairment (i.e., conversion disorder) were excluded. Studies on learning- or behavioral disorders were not excluded in order to include qualitative good comparison studies. The most important study characteristics and results are mentioned in the evidence table (Supplementary Appendix Table 3). Quality assessment of the reviews included in the topics is shown in Supplementary Appendix Table 6 of quality assessment for systematic reviews of diagnostic studies.

Reference test: A reference tests is not yet applicable, as a result of the recent start of diagnostics in CVI in this topic.

A CVI-diagnosis is currently obtained by clinical decision making. In this search, CVI is defined the way it has been defined by the authors of the articles.

Relevant outcome measures:

Reliability, sensitivity, and specificity were used as critical outcome measures by the project group. Outcome measures were not defined *a priori*, the definitions found in the studies were used as outcome measures.

#### Topic Four: Neuro-Radiological Evaluation and Magnetic Resonance Imaging

Question:

Question 4A: How can neuro-radiological examination (imaging) be used to obtain the diagnosis of CVI in children?
Question 4B: Is neuro-radiological examination necessary to obtain a diagnosis of CVI?

MRI examination is chosen because it is the gold standard for children (Sie et al., 2005; Pearce et al., 2012).

To define the search question a systematic literature analysis is performed for the following question.

Two PICO’s have been defined:

First PICO:

P: Patients: Children with a risk of CVI.
I: Investigation: MRI examination.
C: Comparison (control): Other ways to diagnose CVI.
O: Outcome: Prognostic value of MRI for the diagnosis CVI.

Second PICO:

P: Patient: Children with suspected CVI.
I: Investigation: MRI examination.
C: Comparison (control): Children without CVI with MRI or other imaging methods that are also used to investigate CVI.
O: Outcome: MRI findings that are compatible with the diagnosis CVI.

Search and selection:

In Medline (*via* OVID) and Embase (*via* see text footnote 2) in May 2017 and December 2021 with relevant search terms (Supplementary Appendix Figure D) a search has been performed on neuroradiological evaluation and MRI in CVI. The search resulted in 723 hits. Studies were selected on the criteria mentioned in general methods. Studies on acquired damage to the brain such as traumatic brain injury and stroke in children older than 12 years and studies on functional visual impairments were excluded.
  Based on title and abstract 57 studies were selected. After full text reading 48 studies were excluded 9 studies were selected. The study characteristics are shown in the evidence table (Supplementary Appendix Table 4).

#### Topic Five: Genetic Assessment

Question:

Question 5A: What is the position of genetic investigations in obtaining the diagnosis in children in CVI? Is a genetic investigation necessary to obtain the diagnosis of CVI in children?

To define the search question a systematic literature analysis is performed for the following question; Does CVI co-exist in the following genetic disorder?

PICO:

Does CVI co-exist in the following genetic disorder?

P: Patients: children with a developmental age of 18 years or younger.
I: Investigation: A genetic disorder is found.
C: Comparison: Genetic disorder is not found.
O: Outcome: Children with a genetic disorder and CVI.

Search and selection:

In the databases Medline (OVID), Embase (see text footnote 2) in July 2017 a search is performed with relevant search terms for the presence of CVI in genetic diseases. Studies were selected based on the criteria mentioned in general methods; the period for this the search (from 2000) is chosen because of the technical developments in genetics after 2000. The search resulted in 458 hits. Based on title and abstract at first 71 studies were selected of which 68 studies were excluded (case reports and small groups) two studies were selected from the search of another topic, topic two. Five studies were selected. The studies are mainly focused on CVI as part of a genetic disease. Five research outcomes were included in the literature analysis. The most important characteristics and results are mentioned in the evidence table (Supplementary Appendix Table 5).

Relevant outcome measure:

The authors selected the presence of CVI in children with a genetic disorder a relevant critical outcome measure for inclusion.
The authors did not define the outcome measures *a priori* but used the definitions in the studies included.

## Discussion and Organization of Care

A general overview of recommendations is given and an overview of the organization of the diagnostic process for a child with possibility of the diagnosis CVI is given.

This overview is based on the searches in all topics. Organizational consequences of the outcome of the different topics are discussed. Based on the results of the 5 topics, the project group made an inventory of relevant outcome measures for each search question. The project group contained ophthalmologists, a neuropsychologist, a neurologist, a geneticist and a clinical librarian of the Dutch organization of medical specialists. The authors of this study form a subgroup of the project group.

Outcome measures were rated according to their relative importance in recommendations: essential, important (but not essential) and unimportant. The project group prioritized the essential outcome measures based on clinical relevance. This relevance was based on the experience of the project group members and if necessary other specialisms were consulted (neuroradiologist, neonatologist, clinical physicist, child physiotherapist, a representative of child and hospital, a representative of patient association).

After this the project group members approached authors in European countries to make an inventory of organizational possibilities for the diagnostics and referral in CVI. Information was gathered from specialized centers in the United Kingdom, Denmark, Iceland, Norway and Finland, Ireland and Italy on the registration of risk groups (for instance CP and co-morbidity) and on epidemiology of CVI and the diagnostic approach in CVI.

## Results

### Topic One: Medical History and Cerebral Visual Impairment

Question 1A: How should screening on CVI be performed? In this search we focused on medical history.

The studies found could be grouped into three categories (Supplementary Appendix Table 1A):

1 Prenatal causes; Articles dealing with Genetic causes of CVI (congenital malformation of cerebral development; Khan et al., 2007).
2 Perinatal causes and those describing the consequences of pre or perinatal damage and perinatal complications (Solebo et al., 2017), medication during pregnancy (Hamilton et al., 2010; Nadeem et al., 2015) infection during pregnancy (Coats et al., 2000) neonatal hypoglycemia (Yalnizoglu et al., 2007) and studies reporting on postnatal conditions with anoxia or anemia of brain.
3 Postnatal causes; Cranio-cerebral trauma (Poggi et al., 2000), Apparent Life Threatening Event, surgery of the heart with complications (Shen et al., 2009; Bean Jaworski et al., 2018) infections like meningitis (Chaudhary and Sharma, 2012).

Twelve studies were found mentioning risk factors for CVI (see Supplementary Appendix Table 1A): Drugs or medicine *in utero*, intrauterine infections (Cytomegalovirus, CMV), premature delivery/low birth weight, perinatal anoxia, LOW APGAR score, emergency cesarean section, neonatal hydrocephalus, genetic abnormalities, metabolic conditions, cerebral hemorrhages, cerebral infarctions, PVL, intracranial cysts, brain tumors, meningitis or encephalitis, diabetic coma, West syndrome, infantile spasms, and intellectual disability (Wong, 1991; Chen et al., 1992; Houliston et al., 1999; Huo et al., 1999; Shah et al., 2006; Khetpal and Donahue, 2007; Ortibus et al., 2009; van Genderen et al., 2012; Macintyre-Beon et al., 2013; Bosch et al., 2014b; Geldof et al., 2015). By Ventura et al. (2017) a cross-sectional study was performed in 32 children with Congenital Zika Syndrome (CZS). Visual impairment was detected in all 32 infants with CZS (100%). Retinal and/or optic nerve findings were observed in 14 patients (44%). There was no statistical difference between the patients with ocular findings and those without in neurological and neuroimaging abnormalities (*P* = 0.180). All patients (100%) demonstrated these abnormalities; 3 (9%) presented with late-onset of microcephaly. Henderson et al. (2021) characterized visual pathway abnormalities in 105 infants with CZS using Computed Tomography and Magnetic Resonance Imaging (MRI). Overall, 70 of 74 (95%) scans showed occipital volume loss, whereas 9 (12%) showed optic nerve atrophy, 3 (4%) showed chiasmal atrophy, and 1 (1%) showed an ocular calcification.

Question 1B:Which questionnaire should be used in case the professional has a suspicion of CVI, based on the clinical history?

Following the same procedure as for the first question, nine studies were selected (Supplementary Appendix Table 1B) Below we list the questionnaires from these nine studies.

• The PREVIAS (Preverbal Visual Assessment) for 0–24 months (Supplementary Appendix Table 1B) 30 questions in four domains.
• The Scottish Question inventory: Macintyre-Beon questionnaire (Macintyre-Beon et al., 2012).
• The Flemish questionnaire; 46 questions on six domains (Ortibus E. et al., 2011).
• Salavati (Salavati et al., 2017); the CVI motor questionnaire.
• The Express questionnaire: To identify CVI in children born extremely preterm (Hellgren et al., 2020).
• Gorrie (Gorrie et al., 2019): Two questionnaires: the CVI questionnaire (Zihl) and Five questions, derived from the 50 items of the Macintyre-Beon questionnaire as was the HVFQ51 (Higher Visual Function Deficits in Children With CVI) questionnaire (Chandna et al., 2021b).
• The PQCVI (Parental Questionnaire for children with CVI) for children younger than 72 months (Moon et al., 2021).
• The Structured Clinical Question Inventory (SCQI; Philip et al., 2016), based on the questionnaires of Dutton and Houliston (Houliston et al., 1999; Dutton et al., 2010).

Clinometric properties of the different questionnaires were as follows; *Specificity*: The specificity of the PREVIAS was 86,5% for visual attention, 89,5% for visual communication 81,5% for visual-motor coordination and 81,3% for visual processing (Garcia-Ormaechea et al., 2014). The Flemish CVI questionnaire showed a specificity of 70–90% depending on the items included. The motor questionnaire showed a specificity of 96% for GMFCS 1–3 and 98%for GMFCS 4 and 5.

*Sensitivity*: the sensitivity of the PREVIAS was 79% for visual attention, 64% for visual communication, 77,9% for visual motor coordination and 67,5% for visual processing (Garcia-Ormaechea et al., 2014). The sensitivity of the Flemish CVI questionnaire was 0,9 with a sum score of 3.

The CVI motor questionnaire had a sensitivity of 1 for the GMFCS level 1–3 and 97% for the GMFCS level 3–4.

*Accuracy:* The accuracy of the PREVIAS was 83% for visual attention, 77% for visual communication, 80% for visual motor coordination, and 74% for visual processing. The Flemish CVI questionnaire showed an accuracy of 79% (Ortibus E. et al., 2011) for the L94, based on a selection of seven items. The CVI motor questionnaire showed an area under the curve of 99% for GMFCS 1 and 3 and GMFCS 4 and 5.

*Test –retest reliability:* The test-retest reliability was performed with the PREVIAS test, and this was 97% for visual attention, 94% for visual communication, and 98% for visual motor coordination and 98% for visual processing.

For the Macintyre-Beon questionnaire, (Macintyre-Beon et al., 2012) no conclusions can be made on the sensitivity, specificity or accuracy.

The (HVFQ1) study is adapted from the Macintyre-Beon’s questionnaire. One question has been modified, the intra class correlation remains the same (0.98). The reliability of the question they changed is 0.968. By Gorrie et al. (2019) two questionnaire’s validities are studied five questions-sensitivity (81.7%) and specificity (87.2%; ii) CVI questionnaire-sensitivity (96.2%) and specificity-(61.5%).

Rating in levels of evidence:

Question 1A:

**Question 1A:** Which elements does the clinical history need to contain to screen for CVI?

Very low GRADE	Risks for CVI have been identified from literature with the following data. Exposure to drugs or medication during pregnancy (*in utero*), infections during pregnancy (*in utero*), premature birth, very low birth weight, deficiency of oxygen during birth, low APGAR scores, emergency C-section, neonatal hypoglycemia, reanimation, congenital abnormality of brain, microcephaly, hydrocephalus, genetic abnormalities, metabolic disorders, cerebral hemorrhage, cerebral infarctions, PVL, intracranial cysts, tumors of brain, viral or bacterial infections like meningitis or encephalitis, diabetic coma, complications during operation, cerebral palsy, hypotonia, epilepsy (especially West syndrome, infantile spasm) intellectual disability (Wong, 1991; Chen et al., 1992; Houliston et al., 1999; Huo et al., 1999; Shah et al., 2006; Khetpal and Donahue, 2007; Ortibus et al., 2009; van Genderen et al., 2012; Macintyre-Beon et al., 2013; Bosch et al., 2014b; Geldof et al., 2015; Ozturk et al., 2016).

Moderate GRADE	Cortical/CVI may be the most common cause of blindness identified in children with CZS (Ventura et al., 2017). Cortical visual impairment related to structural abnormalities of the occipital cortex is a cause of visual impairment in children with CZS with normal eye examinations (Henderson et al., 2021)

The studies found were all cross-sectional studies or smaller cohort studies, which were mostly based on retrospective analysis. Some studies had a comparative design.

Question 1B:

**Question 1B:** Which questionnaire should be used in case the professional has a suspicion of CVI, based on the clinical history?

Moderate GRADE	The preverbal visual assessment (PREVIAS) has a good specificity a moderate sensitivity and accuracy for the detection of deviation of visual maturation (Lee et al., 2012; Garcia-Ormaechea et al., 2014).

Moderate GRADE	PQCVI (Moon et al., 2021; Parental Questionnaire for Children with CVI) the Cerebral visual function score and the scores corresponding to ventral-stream and dorsal-stream visual functions increased with age. The scores rapidly reached 90% of their maximum values up to the age of 36 months.

Moderate GRADE	The Express questionnaire discriminates well between children born extremely preterm and controls (Hellgren et al., 2020).

Moderate GRADE	The Flemish CVI questionnaire (Ortibus E. et al., 2011) has a moderate accuracy, a good specificity and a moderate sensitivity for the detection of (predominantly) ventral stream deficits. Five Questions and the Flemish CVI questionnaire have good convergent validity, internal consistency and a reliable factor structure (Gorrie et al., 2019).

Moderate GRADE	The SCQI and the CVI motor questionnaire have not been validated with an external reference standard. (Macintyre-Beon et al., 2012; Philip et al., 2016; Salavati et al., 2017)

Moderate GRADE	The HVFQI-51 (Higher Visual Function Deficits in Children with Visual Impairments) can detect a range of HVFDs in children with CVI with good visual acuity and clearly distinguishes these children from typically developing children (Chandna et al., 2021b).

The level of evidence of the PREVIAS is lowered with one level because of limits in extrapolation of the data (bias as a result of indirectness; Supplementary Appendix Table 1B).

The level of evidence of the Flemish CVI questionnaire (Ortibus E. et al., 2011) has been lowered with one level as a result of limits in the design of the study, indistinctness of the independent assessment of the index-test and reference test. However, recently (Gorrie et al., 2019) published good convergent validity, internal consistency and a reliable factor structure for 5 questions, derived from the Macyntire Beon questionnaire (Macintyre-Beon et al., 2012) and the CVI questionnaire. In the list of questionnaires each article has been graded.

Rating in levels of evidence: Rating is related to each paper which has been included.

The following items are advised in the patient history in CVI.

**First** obtain the medical history:

- Structured medical history in a child that is seen because of doubts on its visual functioning (parents concern about visual functioning, or at a regular visit).
- Pregnancy duration (<37 weeks?) and birth weight (<2,000 grams?) APGAR Appearance, Pulse, Grimace, Activity and Respiration score?
- Complications during pregnancy such as infections or medication.
- Complications after birth (such as hypoglycemia, infection) hospitalization (for example cerebral damage by complications during operation, cerebral infection).
- Severe cerebral trauma.
- Developmental milestones (for example delayed motor development or delays in speech, cognition) education (regular or special need).
- Anomalies on neuroimaging (MRI).
- Genetic diagnosis, if available.

**Second**, with regard to the medical history, the questioning with regard to the following items are advised when assessing for CVI:

All questionnaires show a relatively moderate or good sensitivity or specificity, however, for screening all children this is too low, because too many false positive and false negative outcomes are found. We advise to use the questionnaire only in children with a risk on CVI.

- Use the PREVIAS in children younger than 24 months.
- Use the Flemishquestionnaire (Ortibus E. et al., 2011) and the SCQI (Philip et al., 2016) in children of 3 to 5 years of age.
- Refer children that are reviewed due to complications in neonatal care, children with cerebral palsy (GMFCS 1 t/m 5) or developmental delay or traumatic brain injury <12 years to an ophthalmologist at 24 months or when the structured medical history and/or the questionnaire give rise to referral.
- Apply the CVI questionnaires (according to age category) in children that are reviewed due to complications in neonatal care, in children with cerebral palsy (GMFCS 1 t/m 5), developmental delay, traumatic brain injury or stroke <12 years.
- Refer children to an ophthalmologist when the structured medical history and/or the questionnaire warrants a referral sooner/later.

### Topic Two: Ophthalmological and Orthoptic Examinations in Cerebral Visual Impairment

Questions:

Two research questions were defined after discussion with professionals and caretakers who work with children with CVI:

Question 2A: Which are the ophthalmological en orthoptic assessments that are required in case of suspected CVI by ophthalmologist and orthoptist in a general hospital?
Question 2B: Which ophthalmological and orthoptic assessments in CVI can be performed in academic centers in case of suspected CVI?

Description of literature:

Question 2A: Description of studies and results (Supplementary Appendix Table 2A). In a retrospective study, Dutton and Fazzi describe characteristic findings in ophthalmological and orthoptic assessments in CVI (Dutton et al., 1996; Fazzi et al., 2007, 2012) see Supplementary Appendix Table 2A. Fazzi describes lower visual acuity, reduction of contrast sensitivity, abnormal optokinetic nystagmus, reduction of visual field as well as abnormal fixation, reduction of smooth pursuit and saccade quality, strabismus, abnormal eye movements, fundus abnormalities, hyperopia, astigmatism, and myopia. Lowered contrast sensitivity is also found in CVI patients by Mayer et al. (2020) with the DH log_10_ CS test (a new contrast sensitivity test for pediatric patients). More information on visual field reduction was provided by Bosch et al. (2014b). In children with CVI and low visual acuity hemianopia was found in 19%, upper or lower visual field defect in 25% constriction of visual field in 56%.

Tinelli et al. (2020) found a strong correlation between brain lesions and visual function total score (global MRI score *p* = 0.000; hemispheric score *p* = 0.001; and subcortical score *p* = 0.000). Visual acuity, visual field, stereopsis and color were compromised when a cortical damage was present. Ocular motricity (and in particular fixation and saccades) were compromised in presence of subcortical brain damage. Hemispheric severity scores were significantly higher (corresponding to more severe lesions) in children with impaired visual acuity, visual field, stereopsis and color perception. Subcortical score means values were significantly higher in children with an impairment in all the visual items except for nystagmus.

Ruberto et al. (2006) studied OCT and HRT findings in CVI and describes characteristic anatomical findings of the optic nerve: the optic nerve was smaller, the cup/disk ratio larger, the rim smaller and the RNFL thinner in CVI compared to typically developing children. Van der Zee and Evenhuis (2017), see Supplementary Appendix Table 2A, found more crowding deficit in children with CVI compared to typically developing children.

Question 2A: Evidence from literature

**Question 2A:** 

Very low GRADE	Children with CVI seem to have a suboptimal visual acuity and a lower discrimination speed. (Fazzi et al., 2007; Barsingerhorn et al., 2018)

Moderate GRADE	Visual acuity, visual field, stereopsis and color were compromised when a cortical damage was present (Tinelli et al., 2020).

Low GRADE	The ratio between grating visual acuity and crowded visual acuity was significantly higher in children with pathology of the eye and/or brain damage than in typically developing children *A poorer crowding ratio is found in children with CVI compared to normally sighted (healthy) children. A higher crowding ratio was also found in nystagmus and amblyopia* (Huurneman et al., 2012; van der Zee et al., 2017).

Very low GRADE	Children with CVI as a result of cerebral palsy more often seem to have reduced contrast sensitivity (Fazzi et al., 2007, 2012).

Low GRADE	DH log_10_ CS test can be used in children diagnosed with ocular disorders or CVI, Contrast sensitivity was reduced in CVI. Inter-examiner reliability was comparable to that of adults tested previously using the same stimuli and methods (Mayer et al., 2020).

Very low GRADE	Children with CVI seem to show abnormal optokinetic nystagmus more frequently. Strabismus is found more frequently in CVI. Abnormal smooth pursuit movements are found more often in CVI (Fazzi et al., 2007).

Moderate GRADE	Ocular motricity (and in particular fixation and saccades) were compromised in presence of subcortical brain damage (Tinelli et al., 2020).

Very low GRADE	Deviation in saccades are found more often in children with CVI. *Ref* (Fazzi et al., 2012; Barsingerhorn et al., 2019). *Ophthalmological findings that are often found in CVI are strabismus (59%), optic atrophy (42%), nystagmus (12%) and significant refraction anomalies (20%). Visual functions also are deviating in CVI but in 50% of cases, these functions can develop in time* (Khetpal and Donahue, 2007).

Moderate GRADE	In children with CVI optic nerve parameters (OCT, HRT) differ from typically developing children (optic disk surface, cup/disk ratio). (Ruberto et al., 2006)

	With OCT retrograde transsynnaptic (RTSD) degeneration can be detected (Lennartsson et al., 2021) The ganglion cell thinning corresponded with the visual field defects and the extent and location of the primary brain damage. The most important sign of RTSD was asymmetry of the ganglion cell topography within the macular area (Jacobson et al., 2019).

Low GRADE	Children with CVI frequently show visual field abnormalities.; *Hemianopia, upper or lower visual field defect, and constriction of visual field. Ref* (Dutton et al., 1996; Fazzi et al., 2007; Saidkasimova et al., 2007; Luckman et al., 2020).

	In children with CVI the full field peritest (FFP) had best reliability with 44% “good” scores versus 22% for Goldmann perimetry (Portengen et al., 2020).

For this question only observational cross-sectional studies were found, the level of evidence of these studies is low, see also Supplementary Appendix Table 2A. In the studies of Dutton et al. (1996); Fazzi et al. (2007, 2012), and Saidkasimova et al. (2007) there was no control group, for this reason the level of evidence is very low. Tanke et al. (2021) described the Developmental Eye Movement (DEM) test when assessing for CVI. 96 Normally sighted children, 33 visually impaired children and 30 children with CVI were compared. It was found that children with CVI or VI need significantly more time to read the DEM numbers than age-matched controls. Additionally, children with CVI needed more time than children with VI to read the horizontal DEM, but not the vertical DEM.

Rating in levels of evidence: Rating is related to each paper which has been included.

Question 2B: Description of studies and results (Supplementary Appendix Table 2B).

Cavascan et al. (2014) studied 115 children with CVI with sweep VEP. Small inter-ocular differences were found in visual acuity (grating acuity). Clarke et al. (1997) used flash VEP in 44 children, and Frank et al. (1992) studied 60 children with pattern VEP with CVI without ocular pathology, and based on observations. Good et al. (2012) studied 34 children with binocular reduced visual acuity and without ocular pathology with sweep VEP. By Chang and Borchert (2021) visual acuity was assessed clinically in 16 children with CVI using a previously published six-level scale of visual behavior. Grating acuity was assessed by eye tracking using forced-choice preferential looking, this correlated well with clinical visual acuity measurement.

In a systematic review Huurneman et al. (2012) described foveal crowding in children with CVI, two studies were included (Pike et al., 1994; Jacobson et al., 1996); larger effects of crowding were found in CVI than in healthy subjects. Pike et al. (1994) studied visual impairment in 42 children with different lesions on ultrasound examination at 35 weeks gestational age (leukomalacia, large intraventricular hemorrhages, or cerebral infarctions). Predictors of the amount of foveal crowding in children with CVI were: the type of lesions (ischemic lesions are associated with lower visual acuity than hemorrhagic lesions), oculomotor deviations (not being able to fixate), the presence of nystagmus and low visual acuity. Khetpal and Donahue (2007) described the results of ophthalmological examinations in 98 children with CVI; most important ocular findings in CVI were: Exotropia (41%), esotropia (19%), mild optic atrophy (25%), nystagmus (21%), severe optic atrophy (17%), amblyopia (12%), photophobia (4%), and retinal abnormalities (3%). In a study of Sakki et al. (2021) three subgroups of children were described with differentiated visual function characteristics on a gradient of severity. One group showed selective visual perception and visuomotor deficits, the second group showed more severe and broader visual perception and visuomotor deficits, and variable visual acuity and a third group (B), lower-functioning group showed significant visual acuity reduction compared with the first group. Significant group differences were found according to the visual acuity and Hiding Heidi contrast sensitivity categories, with Group B having the lowest scores. The subgrouping method provides the first steps in developing a novel classification system to underpin future clinical diagnostics and profiling of early onset CVI.

Kooiker et al. (2014) performed eye tracking in children with mild oculomotor deviations and children with CVI. A difference in reaction time was found between children with CVI and age matched controls, children with CVI were significantly slower. Lim et al. (2005) used VEP and preferential looking for an assessment in 19 children who were born at term with a history of cerebral damage before or during birth of hypoxia and evidence on MRI for cerebral damage, in a pattern which is typical for hypoxic ischemic encephalopathy. Preferential looking (PL) acuity showed to be lower than normal for this age for nearly all children. Between the first and last measurement the mean increased with one octave; this was also found in acuity VEP. Howes et al. (2022) investigated whether pattern reversal visual evoked potentials (PRVEPs) could predict future visual acuity in infants with CVI. It was found that VEPs in young children with CVI might have prognostic value regarding future acuity.

Skoczenski and Good (2004) showed that grating acuity and Vernier acuity (the ability to discern two lines that do not fuse but run close together) was lowered in children with CVI compared to typically developing children. Graphical research showed that the Vernier acuity was more diminished than the grating acuity and therefore more sensitive in the detection of CVI.

Watson et al. (2007, 2009) investigated 39 children with CVI with sweep VEP (Watson et al., 2009), grating acuity and Vernier acuity and preferential looking. Watson and Haegerstrom-Portnoy (2010) described 33 children with CVI, who were followed for 7 years; the Vernier acuity was more comparable to the PL test than the acuity VEP. Chandna (Chandna et al., 2021a) characterized neural motion processing deficits in children with CVI by using steady-state visual evoked potentials (SSVEP′s) in 31 children with CVI and 28 age-matched healthy controls. Significant deficits in cerebral processing of relative and rotary motion was found but not of absolute motion in children with CVI compared with healthy controls. Vernier acuity, in keeping with good recognition acuity in both groups, was not different, nor were contour-related form responses.

Question 2B: Evidence from literature

**Question 2B:** 

Very low GRADE	The Flash VEP showed a sensitivity that was 85% of the normal VEP in CVI for visual acuity development. The specificity was only 35%. *Ref* (Clarke et al., 1997)

Low GRADE	In VEP stimulation in CVI an abnormal response was found often, especially at the occipital pattern VEP (more than temporo -parietal pattern VEP). *Ref* (Frank et al., 1992)

Moderate GRADE	A subset of patients with CVI have abnormal visual orienting behaviors despite a normal VEP (visuomotor dysfunction; Kelly et al., 2021)

Very low GRADE	Sweep VEP nearly always deviating from normal controls, only in 16% of children with CVI a normal (straight) eye position was found. In CVI usually strabismus is found as well as motility disorders of the eyes. *Ref* (Watson et al., 2007; Watson and Haegerstrom-Portnoy, 2010; Cavascan et al., 2014)

Low GRADE	A sweep VEP showed lower threshold values for grating acuity and contrast sensitivity in children with CVI compared to normal matched controls. *Ref* (Sakai et al., 2002; Lim et al., 2005; Good et al., 2012; Cavascan et al., 2014)

Low GRADE	The Sweep VEP test (Vernier acuity) is useful for prediction of behavioral visual acuity. *Ref* (Watson and Haegerstrom-Portnoy, 2010)

Low GRADE	Grating acuity and vernier acuity was lower in children with CVI than in healthy controls. In a graph vernier acuity was more reduced than grating acuity. Ref (Skoczenski and Good, 2004) *Difference between VEP and Preferential Looking Test is higher in CVI than in normally sighted subjects* (Raja et al., 2021).

Low Grade	With pattern reversal VEP a rough prediction can be made of future visual acuity (Howes et al., 2022).

Low Grade	In the horizontal part of the digital DEM (Developmental Eye Movement Test) children with CVI need more time than VI children of NS children (Tanke et al., 2021).

Low Grade	*Vestibular–Ocular Reflexes are commonly impaired in children with* CVI (Mansukhani et al., 2021).

Very low GRADE	Typical motility disorders of the eye in CVI are paroxysmal ocular deviations (78%), angle strabismus (86%), and reduced coordination of saccades (93%). Orientation in place (spatial; 88%) and fixation (84%) were also reduced. A deviant initiation of saccades and an abnormally preformed saccades were seen the absence of smooth pursuit, abnormal vergence, nystagmus beats and fixation instability were seen as well as problems with systematic orientation in space.*Ref* (Salati et al., 2002).

Moderate GRADE	With Steady-state visual evoked potentials a deficit in processing on more complex relative and rotary motion was found in children with CVI compared to controls, while in processing absolute motion, vernier acuity and contour related form responses no differences were found (Chandna et al., 2021a)

Low GRADE	By the recording of eye movements with eye tracking it was shown that children with CVI reacted significantly slower on visual stimuli (cartoons and movies) than age-matched controls (Kooiker et al., 2014). Eye tracking demonstrates reliability for visual acuity assessment and high correlation with clinical assessment of visual acuity in pediatric CVI (Chang and Borchert, 2021).

Moderate GRADE	Eye tracking in children born very pre-term without apparent White and Gray matter damage on MRI: The infants in the preterm group had longer response times in detecting color patterns (red-green) and motion compared with infants in the comparison group. No impairments were detected in oculomotor functions (saccades, pursuit, and fixations; Pel et al., 2016).

One systematic review was found in which two small observational studies are described that were non-comparative in children with CVI with low level of evidence.

Observational cross-sectional studies were found with low level of evidence. In the studies of Clarke et al. (1997), Sakai et al. (2002); Salati et al. (2002), Lim et al. (2005); Khetpal and Donahue (2007), Watson et al. (2007); Watson and Haegerstrom-Portnoy (2010), and Cavascan et al. (2014) there was no control group, therefore the level of evidence was very low.

Rating in levels of evidence: Rating is related to each paper which has been included.

The following items are advised in an examination for CVI in an academic or diagnostic center;

In Italics the tests that are more specific for CVI;

1 Analyze visual behavior, observe characteristic visual behavior such as overlooking or avoiding (grasping an object while looking the other way, localizing with the periphery of the visual field) or problems with recognition, orientation, perception of movements and simultaneous perception (like overlooking while listening or feeling an object) (Porro et al., 1998).
2 Analyze *motility of the eyes* included *nystagmus*, if present, analyze eye position (strabismus, if present), fixation, *saccades*, pursuit movements and vergence. If possible, *apply eye tracking* to analyze eye movement disorders.
3 Analyze head position and torticollis.
4 Analyze *pupil and pupil reflex* and anisocoria and RAPD. A deviation in pupillary response can be a sign of optic nerve problems or pathology of chiasma (Naber et al., 2018). Pupillary responses are often normal in CVI even if mild bilateral optic atrophy is found. This part of the examination is also important in differential diagnosis.
5 Analyze binocular vision (stereopsis).
6 Measure visual acuity (matching or verbal test) near and at distance (for instance ETDRS, illiterate E, numbers, Landolt C, LH charts) and measure *the crowding ratio at near* binocular (Huurneman et al., 2012) and if possible, also monocular. A crowding ratio of 1 is found in typically developing children of about 7 years old, which means that there is no crowding deficit after 7 years. In visually impaired children and children with CVI, this reduction of crowding was not found.
7 If visual acuity measurement at distance is not found or if it is not possible, try to measure visual acuity non-verbally for each eye and for both eyes for instance with Teller Acuity Cards (TAC) or Cardiff Acuity Test.
8 Measure the *contrast sensitivity* verbally or non-verbally. For instance, with Vistech or Hiding Heidi in younger children, monocular and binocular. A reduced contrast sensitivity can be found in CVI.
9 *Measure accommodation* with dynamic skiascopy or another measurement method.
10 Investigate *visual fields* with a confrontative measurement (Donders method or behavioral method such as Stycar balls, the Behavioral Visual Field Testing (BEFIE test), the double arc perimetry or equivalent test when standard methods could not be performed (Koenraad, 2016).
11 If possible, depending on the developmental age, make a visual field with standard perimetry Goldmann and/or automatic perimetry if possible.
12 Analyze the anterior segment and media with a slit lamp (if no data are obtained before).
13 Obtain refraction in mydriasis (cycloplegia) if no data have been obtained before.
14 Analyze the optic media and *fundus by ophthalmoscopy* in mydriasis.
15 Make *OCT scan* of the optic nerve and macula with RNFL analysis and Ganglion cell layer (GCL) analysis. In CVI the optic nerve can be smaller or a larger excavation is seen (Ruberto et al., 2006; Jacobson et al., 2019).
16 If visual acuity has not been measured adequately, observe *optokinetic nystagmus*.
17 If visual acuity has not been measured adequately and OCT does not provide enough information on the optic nerve, *consider VEP*.

All items are meant to investigate CVI; deviations in fixation, saccades, accommodation, crowding ratio at near, visual field, and optic nerve are of specific importance for CVI.

Besides the ophthalmological and orthoptic examination as performed in academic centers there are two important issues for CVI.

1 Multidisciplinary cooperation is necessary. The multidisciplinary team for CVI diagnostics should minimally contain a pediatric ophthalmologist, pediatric neurologist, orthoptist or optometrist, neuropsychologist. After ophthalmological and orthoptic examination the ophthalmologist should organize multidisciplinary meetings to discuss the results and the possibilities for further assessments, if necessary. For instance, the ophthalmologist should discuss with the neurologist (or pediatrician with specialization in neurology) referral criteria if a neurological diagnosis is suspected or a neurodegenerative diagnosis is in the differential diagnosis.
2 The academic (pediatric) ophthalmologist can refer the child to a rehabilitation center or a specialized center or school for visually impaired children if there is evidence of CVI and the etiology is clear.

### Topic Three: Neuropsychological Assessment

Questions:

Question 3: Which neuropsychological tests can be used in the neuropsychological examination of children with CVI?

Question 3: Description of studies and results (Supplementary Appendix Table 3).

**Question 3A:** 

Low GRADE	The Motor-free Visual Perception Test and the Developmental Test for Visual Perception seem to have a high internal consistency for the complete test (Auld et al., 2011)

Low GRADE	Eyetracking for gaze fixation duration and saccades. Children with visual impairments due to cerebral damage show weaker Gestalt perception and had different looking patterns than children with ocular or without visual impairments. Children with CVI and brain damage performed significantly worse on the animate items than the group without brain damage (Van der Zee et al., 2019) To study the selectivity of visual perceptual impairment in children with early brain injury, the L94can be administered. It discriminates between congenitally disabled children with and without risk for CVI (Stiers et al., 2001)

Moderate GRADE	The CVIT specifically measures CVI-related visual perception deficits and is not mediated by intellectual abilities or low visual acuity (Vancleef et al., 2020)

Moderate GRADE	The most discriminating dimensions between CVI and no CVI were object/picture recognition (*r* = 0.56), visual spatial perception (*r* = 0.52), visual discrimination and matching (*r* = 0.47), and figure-ground perception (*r* = 0.39; Ben Itzhak et al., 2021b).

A systematic review (Auld et al., 2011) describes the psychometric properties of assessment instruments for visual perception in children with hemiplegia. Because of the presence of CVI in a majority of children with cerebral palsy (CP)/hemiplegia this article is included in the literature analysis. Children with cerebral palsy (CP) often present with CVI up to 50% in an overview study by Ego et al. (2015) and more than 90% of children with CVI have multiple impairments (Khetpal and Donahue, 2007), including severe motor and language disorders. Ego described a systematic literature review to assess the frequency of visual–perceptual impairment and its relationship with patient characteristics. The systematic analysis (Auld et al., 2011) found 3 assessment instruments that met the inclusion criteria: the Motor Free Visual Perception test (MVPT), Test of Visual Perceptual skills (TVPS), and the Developmental Test of Visual Perception second edition (DTVP-2). For all tests psychometric data are available of normative studies, but not of studies with children with hemiplegia.

Vancleef et al. (2020) measured CVI related visual perception deficits with the Children’s Visual Impairment Test, 3–6 years (CVIT3–6). The CVIT 3–6 is grounded in knowledge of visual perception. Reliability was assessed via test-retest correlation and intraclass correlation coefficient (ICC) in typically developing children, children with CVI, intellectual impairment, and simulated impaired vision (validation groups *n* = 59, mean developmental age = 4 year 10 month, 27 females, 32 males).

Ben Itzhak et al. (2021b) assessed the clinical records of 630 children (median age 77 months, interquartile range 63–98 months) referred to a CVI clinic and evaluated the diagnostic value of seven predefined dimensions of a visual perceptual schema: visual discrimination and matching, object or picture recognition, visual spatial perception, figure-ground perception, motion perception, visual short-term memory, and scene perception, using the combined results of a set of 23 subtests derived from the Beery Visual-Motor Integration test (VMI), TVPS, L94, Revised Amsterdam Children’s Intelligence Test (RAKIT-2), Preschool Judgment of Line Orientation (PJLO), Neuropsychological Assessment (NEPSY-II) and several motion perception tasks.

Van der Zee et al. (2019) studied Gestalt perception using images of the Kaufman Assessment Battery for children’s subtest Gestalt Closure. They analyzed gaze behavior recorded by eye tracking of 20 children with CVI.

#### Results

Specificity and Sensitivity for CVI were reported for the dimensions of the visual perceptive schema of Ben Itzhak et al. (2021b). For object/picture recognition, a good area under curve (AUC) was found (AUC 83%, sensitivity 82%, specificity 69%; fair AUCs were found for visual spatial perception 80%, visual discrimination and matching 78%, figure-ground perception 73%; sensitivity ranging from 75 to 78% and specificity ranging from 65 to 67%. Motion perception and visual short-term memory showed poor AUCs 63%, sensitivity 71–73% and specificity 45–49%.

Test-retest reliability is high for the complete MVPT in normally developed children and children with learning problems (ICCs = 0,63–0,79). For the TVPS, test-retest reliability is also high in children with learning problems (ICC = 0.81). No ICCs for the DTVP were reported. Internal consistency is high for the MVPT (Cronbach’s coefficient α 4–10 years = 0,69–0,87 > 11 years: 0,86–0,9). For the TVPS, studies report varying internal consistency (low-excellent). The DTVP shows a high internal consistency (Cronbach’s α coefficient-0,8–0,97 concerning the subtests; 0,93 or higher for the complete test).

For the CVIT3–6, high correlations were found with tests with a strong visual perception component (L94: *r* = 0.74, *p* < 0.001; SON-R 2.5–7: *r* = 0.37, *p* = 0.01) and low correlations with other tests (Beery-VMI: *r* = 0.25, *p* = 0.09; SRS: *r* = 0–0.26, *p* = 0.09). Lowest scores were observed for children with CVI compared to the other validation groups [F(3,44) = 5.1, *p* = 0.003], supporting its validity. The tool specifically measures CVI-related visual perception deficits and is not mediated by intellectual abilities or low visual acuity. Good test-retest reliability (*r* = 0.82, *p* < 0.001, ICC = 0.80) was found (Vancleef et al., 2020).

The most discriminating dimensions between CVI and no CVI were Object/picture recognition (*r* = 0.56), visual spatial perception (*r* = 0.52), visual discrimination and matching (*r* = 0.47), and figure-ground perception (*r* = 0.39; Ben Itzhak et al., 2021b). Gestalt perception was worse in children with CVI as compared to visual impaired children with ocular disorders (Van der Zee et al., 2019). Children with CVI performed extremely weak, i.e., 60% scored below the level of the weakest 9% of healthy controls.

Given the lack of evidence for the diagnostic value of individual tests for visual perception, an assessment protocol could be composed of the neuropsychological subtests as indicated by the Delphi study reported by Ben Itzhak and derived from the tests MVPT, DTVP, TVPS, L94, Beery VMI, RAKIT-2, PJLO, and NEPSY-II.

• Dimension Object/picture recognition: L94 De Vos-task, Visual matching, Line drawings occluded by noise, Overlapping line drawings, Unconventional object views, RAKIT-2 Figure recognition, Hidden figures, Motion-defined form, and Biological motion.
• Dimension Visual spatial perception: Beery VMI Perception, Motor-coordination, visual-motor integration, TVPS-3: Visual discrimination, Visual spatial relationships, Visual memory, Visual sequential memory, Visual figure-ground, Visual closure, L94 De Vos-task, and Visual matching.
• Dimension Visual discrimination and matching: Beery VMI Perception, TVPS-3: Visual discrimination, Visual spatial relationships, Visual memory, Visual sequential memory, Visual figure-ground, Visual closure, L94 De Vos-task, Visual matching Overlapping line drawings, RAKIT-2 Hidden figures, and PJLO.
• Dimension Figure-ground perception: TVPS-3: Visual figure-ground, Form constancy, L94 Line drawings occluded by noise, Overlapping line drawings, RAKIT-2 Hidden figures, and Motion defined form.

Given the wide variety of available subtests, the project group advises to adjust assessment protocols to the child’s age-level and mental abilities.

Rating in levels of evidence: Rating is related to each paper which has been included.

#### Screening and Diagnostic Assessment

If deficits in visual attention or visual perception are suspected, screening of visual perceptive functioning with the Motor-free Visual perception test is recommended and can be directive for further assessment. Refer for further assessment if test outcomes are typical for CVI. Referral can also be considered if MVPT results are normal since it does not measure all aspects of visual perceptive functioning. Refer for neuropsychological assessment for CVI to a specialized center if the neurocognitive profile shows discrepancies in disadvantage of the level of visual processing (i.e., visual selective attention, visual perception, visual memory, or visual processing speed).

Diagnostic assessment. The following assessment is advised in a child with possible CVI. Conduct neuropsychological assessment in children suspected of deficits in visual attention or visual perception using the following neuropsychological tests and principles:

- Assess visual perception with the DTVP, TVPS, L94, and CVIT 3–6. Recommendations for a fixed set of tests is not supported by evidence. The studies indicate several visual-perceptive dimensions that are sensitive to CVI, that, however, consist of the combined results of neuropsychological tests or subtests.
- It is therefore advised to select subtests from the reported set of tests and adjust the protocol to the age-level and mental abilities of the child.
- In addition the RAKIT-2, PJLO, NEPSY-II, Beery VMI, and Motion perception tasks can be used. Neuropsychological assessment of disorders in object/picture recognition, visual spatial perception, visual discrimination and matching, figure-ground perception, and visual organization or recognition in visual noise. There is no preferred test based on the literature.
- Also assess other aspects of visual information processing: Visual selective attention, visuo-spatial perception (global versus local attention).
- Discrepancies of the neurocognitive profile indicating weaknesses of visual information processing within the domains of attention, motor, and memory functions.

Furthermore the project group recommends to compare test results with normative data matched on mental developmental age. Verify whether disorders in visual attention and perception are associated with the medical history and the limitations reported in activity and participation in daily life.

Discuss symptoms of other developmental disorders and/or differential diagnostic considerations with parents and caretakers of children suspected of CVI. In very young children and in case of doubt of the validity of assessment outcome, consider temporally using a “working diagnosis” CVI to provide access to care facilities, if needed. Plan follow-up assessment is case CVI has been diagnosed or if there are doubts about the exact type of dysfunction in visual attention or perception, until a developmental age of 12 years, or at least until no change in the diagnostic outcome is expected. Discuss the outcome of neuropsychological assessment in a multidisciplinary team and incorporate the findings in diagnostic decision making and follow up trajectory.

### Topic Four: Neuro-Radiological Evaluation and Magnetic Resonance Imaging

Questions:

Question 4A: How can neuro-radiological examination (imaging) be used to obtain a diagnosis of CVI in children?
  Question 4B: Is neuro-radiological examination necessary to obtain a diagnosis of CVI?

Description of literature: Question 4 AB: Description of studies and results (Supplementary Appendix Table 4).

**Question 4AB:** 

Very low GRADE	In children with CVI, MRI abnormalities were often found related to the visual pathways. The abnormalities are not identical. In children who have no CVI abnormalities on MRI are found less often. (Lambert et al., 1987; Uggetti et al., 1996; Sie et al., 2005; Khetpal and Donahue, 2007; van Genderen et al., 2012)

Moderate GRADE	Periventricular leucomalacia on MRI was found to have a strong association with CVI in all 30 studies. Only 13 (43%) studies described dorsal and/ventral stream dysfunction (Philip et al., 2020).

Low GRADE	MR imaging showed signs of cortical dysgenesis leading to congenital brain malformations such as polymicrogyria consistent with a prenatal timing of CNS injury (Ho et al., 2020).

Moderate GRADE	CVI participants have a significantly higher mean motion coherence threshold (determined using a random dot kinematogram pattern simulating optic flow motion) compared to controls. Using functional magnetic resonance imaging (fMRI; Pamir et al., 2021).

Moderate GRADE	Brain lesion severity strongly correlated with visual function total score. Moreover, visual acuity, visual field, stereopsis and color were compromised when a cortical damage was present, while ocular motricity (and in particular fixation and saccades) were compromised in presence of subcortical brain damage (Tinelli et al., 2020).

In 53 premature babies with PVL a correlation was found between the presence and amount of damage to the occipital white and gray matter and CVI (Sie et al., 2005). MRI images were obtained at a mean age of 20 days after birth and at 1,5 years Khetpal studied 98 children retrospectively (Khetpal and Donahue, 2007) with final diagnosis CVI, in 70% an MRI was made of which abnormalities were found in 83%. Lambert et al. (1987) included 75 children younger than 6 years with hypoxic seizure. CT scan was made in 28 children MRI in two children. In these children (all with diagnosis CVI) global atrophy was found in 12 children, PVL in 8 children, an infarction of the occipital lobe in and mild atrophy of the optic nerve in 6 children and watershed area infarction of the parieto-occipital lobe in 2 children. The 2 children with normal scans had good visual outcome. Uggetti et al. (1996) studied 27 premature children with cerebral palsy as a result of periventricular leucomalacia, 17 were diagnosed with CVI. The MRI findings corresponded with visual acuity. There was a relation between visual functions and a reduction of the peri-trigonal white matter and calcarin atrophy contra laterally.

In a retrospective study 53 schoolchildren were described by van Genderen et al. (2012) with good binocular visual acuity but with suspected CVI. They were referred to a center for rehabilitation. The 30 children with diagnosis CVI (based on orthoptic and ophthalmological examination) had an abnormal medical history, 21 were born prematurely, 7 had perinatal asphyxia two children had trauma capitis, 20 children had cerebral palsy and 8 had epileptic seizures. In this group abnormalities on MRI were found in 86% of children. In the 27 children who were not diagnosed with CVI 6 children (25%) had an abnormal medical history; prematurity in 4 children, hydrocephalus in one child, epileptic seizures in one child.

Magnetic resonance imaging abnormalities. MRI abnormalities can also be found in genetic anomalies of brain or congenital infections like CZS (CZS) or congenital cytomegaly virus (CMV); (Bosch et al., 2014b; Henderson et al., 2021).

In a review, (Philip et al., 2020) studied the relationship between brain structure and CVI in children with Cerebral Palsy. Based on the outcome of the search they reported the brain lesion on MRI linked to CP and CVI thus grouping the subjects into the brain maldevelopment group, the white matter lesion group, the gray matter lesion group, or the postnatal lesion group and a normal MRI group.

1 Brain maldevelopment (*lesions that occurred in the 1st and 2nd trimester*) accounting for 3% of all brain lesions. In this group, spastic subtype of CP was the most common presentation (84%, *n* = 103 subjects).
2 White matter lesions (*those occurring in early 3rd trimester or preterm babies*) were the most common (83.5%, *n* = 665 subjects) classified brain lesion on MRI. Periventricular leucomalacia was the most common MRI finding reported followed by ventricular dilation, thinning of the corpus callosum and gliosis of the occipital area. CVI was reported in all the 30 studies, with dorsal stream dysfunction described in 9 studies.
3 Gray matter lesions (*occurring in the late 3rd trimester of gestation or term*) accounted for only 4% of all classified brain lesions with spastic CP seen in 103 subjects and non-spastic CP in 16 subjects.
4 The fourth and the least common pattern of brain lesions are *those that occurred in the post-natal period* and accounted for 1.0% (*n* = 8) of all the brain lesions.

In 13 of the 30 studies, CVI was further characterized into ventral or dorsal stream function or both. (i) Ventral stream (occipito-temporal pathway): also called “what” (perceptual) pathway, connecting the occipital lobe to the temporal lobe and involved with orientation, visual memory and recognition of faces, shapes, objects, and words; (ii) dorsal stream (occipito-parietal pathway): also called “where” pathway, connecting the occipital lobe to the posterior parietal lobe and is responsible for subconscious mapping of the visual scene, triggering the frontal eye fields to bring about quick eye and head movement to the object of regard subserving visual attention movement and handling of complex visual scene (Milner and Goodale, 2006).

Tinelli et al. (2020) studied 72 children with PVL (periventricular leucomalacia), and found a significant correlation between brain lesion severity and visual function impairment in children with PVL. In a case control study (Pamir et al., 2021) compared 12 CVI children (17 years) with 18 controls (19 years). They studied activation response profiles in functionally defined early (i.e., primary visual cortex; area V1) and higher order (i.e. middle temporal cortex; area hMT+) stages of motion processing. Mean motion coherence threshold for CVI participants (44.18% ± 23.3 SD) was significantly higher (i.e., indicative of worse performance) compared to controls. Specifically, increased motion coherence was associated with an increased BOLD signal response in area hMT + for CVI, but not for the control group.

Rating in levels of evidence: Rating is related to each paper which has been included.

The following steps are advised in a child with possible CVI.

Always plan a consultation of a pediatric neurologist to organize MRI in CVI.

Discuss the implications of MRI with parents or caretakers because of the burden and risk for the child. Also, it is advised to relativize the outcome of MRI for the diagnosis CVI.

Consider MRI when there are doubts about CVI, this is necessary to exclude other causes of visual impairment. MRI can give more information on the etiology of CVI. Structural and functional neuroimaging approaches in CVI can help gain insight into abnormalities of white matter connectivity and cortical activation patterns, respectively (Merabet et al., 2017).

Discuss the results of MRI or ultrasound (in children younger than 1 year old) in CVI in a multidisciplinary team and take the considerations of different disciplines into account for diagnostic follow up. Potentially, MRI would be the best imaging modality to assess neonatal brain damage, but requires infant displacement from the neonatal intensive care unit (Sie et al., 2005), the additional value of early MRI as compared to cranial ultrasound was described previously. It was shown that early MRI can detect the precise site and extent of white matter injury at an earlier stage and allows a better differentiation of lesions than ultrasound.

In CVI ophthalmological and orthoptic examinations applied in academic centers assessment can be accompanied by pediatric, neurological, neuropsychological and neuroradiological examinations. Therefore, a multidisciplinary CVI team is necessary, this team may also function between different (specialized) centers.

### Topic Five: Genetic Assessment

Questions:

Question 5 What is the role of the genetic diagnosis in the referral of a child for clinical investigation for CVI?

Description of literature:

Question 5: Description of studies and results (Supplementary Appendix Table 5).

**Question 5:** 

Low GRADE	The following genetic disorders seem to be associated with CVI: Down syndrome (trisomie 21), 1p36 deletion syndrome, 17p13.3 deletion syndrome (Miller-Dieker syndrome), 22q13.3 deletion syndrome (Phelan-McDermid syndrome), CDG type 1a, complex I deficiency, Lissencephaly (gene DCX), Pelizaeus-Merzbacher syndrome (gene PLP1), atypical Rett syndrome (gene CDKL5), NGLY1, AHDC1, NR2F1, PGAP1and tuberous sclerosis. (Bosch et al., 2014a,b, 2016; Wan et al., 2019; Wilton et al., 2021)

The evidence from the literature for presence of CVI in children with a genetic disease is lowered with two levels to “low” as a result of the draw backs in the study design and lack of information about the control group and the low number of patients.

Rating in levels of evidence: Rating is related to each paper which has been included.

In all but five studies, a possible association between CVI and a genetic cause was found in case reports or case series. In general congenital anomalies of brain can give CVI.

Research on genetic causes in a CVI population is only found in five studies (Bosch et al., 2014a,b, 2016) and only in persons with a visual acuity of ≤6/18. There are no studies with large populations of persons with a specific genetic disorder that have been investigated on presence or absence of CVI. Behavioral features of CVI are common in children with Down syndrome 38% of parents reported CVI symptoms (visual perception difficulties; Wilton et al., 2021). Children with Down syndrome have significantly lower visual acuity than typically developing children at distance and at near that cannot be corrected by distant refraction. They often present with accommodation deficit, crowding and strabismus (de Weger et al., 2021) and need bifocals to correct this (de Weger et al., 2019).

In several case series and reports, CVI was mentioned as one of the features. Although these reports are not of sufficient quality to include in the guidelines, a table is made of genetic diagnosis found if one of the features mentioned was CVI (Supplementary Appendix Table 5: Genetic cause found in case reports and case series).

The following remarks must be made:

- Cerebral visual impairment can be co-morbidity, without a causal relation, in a child with a genetic disorder.
- The table does not provide information on frequencies of CVI found in this diagnosis and if referral for further investigation on CVI is justified.
- Absence of a genetic disease in the table does not mean CVI cannot be found in this disease, because a structural examination on specific CVI items is not always performed or not mentioned in the title or abstract.

## Discussion

### General Discussion and Organization of Care in Cerebral Visual Impairment

Based on the results of the five topics, the project group made an inventory of relevant outcome measures for each search question. Outcome measures were rated according to their relative importance in recommendations: Essential, important (but not essential), and unimportant. The project group prioritized the essential outcome measures based on clinical relevance.

Next the project group members approached authors in European countries in order to make an inventory of organizational possibilities for the diagnostics and referral in CVI.

All aspects of the care in order to diagnose and refer in CVI such as coordination, communication, financial means, human resources, and infrastructure are concerned in the start of the care for CVI. Because of the diversity in CVI, the care is complex and difficult to specify. Explanation of how the diagnosis and referral of CVI is organized in different European countries, is integrated in this chapter. Organizational and financial interferences were signaled and possible solutions described (Francke et al., 2008).

### Topic One: Medical History and Cerebral Visual Impairment (Search Question 1A,B)

#### Medical History

The structured medical history is related to the general categories which can be defined for children with risks on CVI as a result of congenital anomalies of brain or events pre-peri or postnatal that lead to brain damage during cerebral development.

None of the questionnaires offers sufficient screening in all CVI domains or for all age groups. In addition, children with behavior or emotional problems have a higher risk on false positive results with the current available questionnaires (Geldof et al., 2015).

There are two ways in which children with possible CVI can start in the diagnostic chain:

1 Parents are signaling deviant viewing behavior and are consulting a general physician, pediatrician or youth doctor or ophthalmologist. In this case a structured medical history (and if necessary a questionnaire) can be applied.
2 Identifying children by a questionnaire by healthcare professionals, this can be organized when risk groups are defined.

The use of a questionnaire is recommended, specially in high risk groups. A questionnaire provides a semi-structured interview about visual acuity and visual behavior. Some questionnaires clearly have an added value in screening when used during a first visit with a caretaker.

High risk groups for CVI:

Children with events during brain development; Prenatal; Perinatal; Postnatal (Flanagan et al., 2003; Rahi and Cable, 2003; Hellgren et al., 2016; Hellström et al., 2018; Philip et al., 2020); (group I and II) Children with congenital anomalies of brain (group II and III).
Children that are at risk for CVI on a basis of their history should have a structured history taken. If there are risk factors found, a questionnaire on CVI can be applied.
A referral at 24 months of age is always indicated for.
I Children who are included in the follow up group for Neonatal Care; prematurity < 30 weeks and/or birth weight < 1,000 gram.
II Children with Cerebral Palsy (GMFCS 1 t/m 5).
III Children with a developmental delay or TBI (traumatic brain injury).
In these groups, a CVI questionnaire is always indicated.

Group I pregnancy < 30 weeks and/or birth weight < 1,000 gram:

Based on the classification system, which was made for research goals, (Geldof et al., 2015) has found 3,8 cases of CVI compared to one in the control group, this is 24% of children in a group born after 32 weeks of pregnancy and/or with a birth weight < 1,500 grams. Based on this research it is not possible to define in how many children the diagnosis CVI was finally made. At 5 years of age, the results were evaluated, in the group of children diagnosed with CVI there was no need for rehabilitation.
In case of prematurity (Pregnancy < 37 weeks and/or Birth weight < 2,000 grams) and/or in case of clear evidence for CVI based on the structured medical history or the CVI questionnaires, it is important to refer the child to an ophthalmologist. Because of the multifactorial cause of CVI, it is not possible to formulate clear cut refer criteria based on the history or the CVI questionnaires. In Sweden 1–2% of children with ROP and prematurity (<27 weeks and/or <1,500 gram) had CVI and low visual acuity (VA < 6/18)(Larsson et al., 2005; Holmström et al., 2014). These studies were not included in the search because ROP was the primary goal of the study and CVI was not mentioned in the abstract. As a result, the search did not specifically include CVI and subnormal visual acuity is missed. Therefore, it is possible that the mentioned numbers are an underestimation. The lower the birth weight and the shorter pregnancy duration the higher the risk of CVI (Jacobson and Dutton, 2000).
Although there are just a few studies, the guideline-project group is convinced that children with severe prematurity should all be screened on CVI.
A birth weight of 1500 grams or less is classified as severe prematurity.
However, neonatologists (Federatie medisch specialisten, 2015) advise a follow up for children born after 30 weeks and/or birth weight < 1,000 gram.

Group II cerebral palsy:

Children with cerebral palsy are at risk for CVI. Visual problems are seen more often in children with spastic quadriplegia than in children with spastic diplegia. In a review Colver (Colver et al., 2014) described visual problems (ocular pathology and CVI) in 35% of the children; therefore, ophthalmological examination is advised on a regular basis in all the children with cerebral palsy. The classification system in cerebral palsy Gross Motor functioning Classification System (GMFCS) consists of five classes, from 1 (less impaired to 5 most impaired).
For children with GMFCS 1 t/m 5 screening by a questionnaire is proposed.
Children who incur a cerebral trauma after the age of 2 years (traumatic or non-traumatic) are at risk for CVI as well (Kivlin et al., 2000; Philip and Dutton, 2014). Although there are no guidelines for this group, we advise screening of visual functions by means of a questionnaire. Depending on the type of trauma and the developmental age the PREVIAS (preverbal visual assessment) CVI Questionnaire (Ortibus E. et al., 2011) or the motor questionnaire (Salavati et al., 2017) can be used.

Group III Developmental delay:

Children with developmental delay (independent of the cause) are at risk for CVI. In 5–20% of these children with intellectual disability, CVI and a low visual acuity is found (Warburg, 2001; van Isterdael et al., 2006; Nielsen et al., 2007). For this group we advise screening with CVI screenings lists and questionnaires.
Children older than 5 years, where there were previously no doubts about visual functioning and without risk factors for CVI can still have visual problems. It is therefore important for the professional caretaker to take a structured medical history and apply the CVI questionnaire when there are doubts on visual development.
Although the Flemish CVI questionnaire shows a good AUC curve based on 7 selected items, the questionnaire has been studied in relation with a set of object recognition items with visual-spatial elements and attention specific items (dorsal stream items). In the research population, no children with only dorsal stream problems were included. The age-range of the included children was limited, only children with a developmental age between 2,75–6,5 years were included. New evidence for this questionnaire was published by Gorrie et al. (2019) and Ben Itzhak et al. (2020). None of the questionnaires offers sufficient screening in all CVI domains or for all age groups.

### Topic Two: Ophthalmological and Orthoptic Assessment (Search Question 2A,B)

It is important to differentiate CVI from other diagnoses that resemble CVI, such as visual impairment by ophthalmological causes, delayed visual maturation, optic hypoplasia, congenital and acquired deviations of the optic nerve, hereditary retinal abnormalities, tumors, or other causes of compression of the optic nerve and visual pathways.

The diagnosis of children with CVI could be accomplished in academic or other specialized centers. A major problem is that diagnosing CVI is not included in the training of ophthalmologists. Another problem is that daily practice in ophthalmology is based on quick visits to the ophthalmologist. Children with CVI do not fit into this system because several assessments are needed. And the different assessments mentioned all add to the diagnosis. Priority can be given to the more specific tests in CVI in ophthalmological and orthoptic examination in this guideline (in italics) regardless of developmental age.

If an ophthalmologist has doubts about the diagnosis CVI and/or about the etiology, the child can be referred to an academic center for more specialized ophthalmological and orthoptic examinations when one of the following results is found in combination with a history that is indicative for possible CVI.

*A: binocular suboptimal visual acuity, pupil and pupil reflex, insufficient accommodation and/or a crowding ratio at near that is too high for the calendar age without an explanation such as amblyopia.*
*B: abnormal findings of the optic nerve, such as: partial or total pallor, hypoplasia, a too large excavation, without any other explanation (such as genetic optic atrophy).*
*C: anomaly in saccades (delay) and or fixation (fixation area), following movements and or vergence.*
*D: visual field defect, reduction of contrast sensitivity.*
*E: If available OCT, VEP and Eye tracking are helpful.*

The outcome of the ophthalmological and orthoptic examination can be:

A: Progressive cause of brain dysfunction; Referral to a pediatric neurologist or a specialized pediatrician.
B: There are doubts about the etiology and/or the diagnosis CVI. Refer the child for neurological or genetic assessment.
C: Cerebral visual impairment is diagnosed or suspected with a clear etiological factor (congenital abnormality of brain or, perinatal or postnatal brain damage); children can be referred to a center for rehabilitation of visual impaired or blind people.

A diagnostic routing should be defined with neurological, neuropsychological, and genetic examinations. After this possible additional assessments or rehabilitation can be discussed in a multidisciplinary meeting with parents and medical specialists.

The ophthalmologist can coordinate this process and is in charge of the diagnostic chain in case of CVI. The ophthalmologists can discuss the findings with the different professionals (orthoptist, child neurologist, neuropsychologist, neuroradiologist, clinical geneticist, child physiotherapist). Intensive dialog with a pediatric neurologist is recommended.

The diagnostic process of CVI is complicated and needs a follow up of at least 2 years, depending on the developmental age at referral.

### Topic Three: Neuropsychological Assessment

*Five neuropsychological tests were found with low to moderate evidence for diagnostic accuracy for CVI, the MVPT, DTVP, TVPS, L94 and the CVIT 3–6.* Based on the psychometric properties, the MVPT is preferred for screening of visual perception and the DVTP, TVPS, L94 and CVIT 3–6 are preferred in the diagnostic assessment of CVI, because its subtests differentiate several aspects of visual perception. Also, the use of several other neuropsychological (sub)tests have been studied, but evidence for the diagnostic value of individual tests is lacking and most tests seem involved in multiple visual perceptive dimensions (Ben Itzhak et al., 2021b). Since performance differences on the DVTP were found between American and Asian children (Chan and Chow, 2005; Cheung et al., 2005), adaptation of normative data to local populations seems required. In addition, disorders in visual organization, in recognition in reduced circumstances and recognition in noise are likely typical disorders of visual processing in CVI (Stiers et al., 2001).

The project group advises to estimate the level of mental development before assessing visual perception to avoid false positive assessment outcome and over-diagnosing CVI (Stiers et al., 2001). Follow-up of the neuropsychological assessment is recommended until 12 years of age, because of continuous cerebral development and subsequent development of perceptive visual functioning (Kovacs, 2000; Klaver et al., 2011).

Despite limited evidence for the reliability and validity of neuropsychological tests for the assessment of CVI, it has been recommended to assess a wide range of visual perceptive functions (Fazzi et al., 2009; Boot et al., 2010; Ortibus E. L. et al., 2011). This is also recommended in models for neuropsychological assessment of visual processing functions derived from clinical practice, indicating assessment of visual selective attention, visual-spatial perception, visual identification, visuomotor functions, visual (working)memory, and visual processing speed (Fazzi et al., 2009; Zuidhoek, 2015; Zuidhoek et al., 2015). Furthermore, to indicate CVI, abnormal test results should be differentiated from disorders in visual sensory and oculomotor functions, executive, attention, memory, mental or auditory functioning to indicate CVI. Hence examining the existence of such potentially co-occurring dysfunctions could be necessary. Disorders in visual organization, recognition in less optimal conditions and recognition in visual noise seem typical for CVI may guide interpretation of the neuropsychological assessment of CVI (Stiers et al., 2001).

Developmental disorders that could incorporate visual perceptive dysfunctions need to be considered during diagnostic decision-making. Visual dysfunctions found in children with developmental disorders include dysfunctions in visual attention, visual processing speed or visual memory (e.g., in ADHD and autism spectrum disorder), dysfunctions in visual identification (dyslexia) dysfunctions in visual spatial perception, visual-motor coordination (developmental coordination disorder). However, the comorbidity of CVI and developmental disorders has not been studied sufficiently (Grinter et al., 2010). Identified visual dysfunctions should therefore be evaluated in a multidisciplinary setting. Thereby, neuropsychological assessment can contribute to both accurate diagnostic decision-making as well as identifying to therapeutic possibilities.

### Topic Four: Neuro-Radiological Evaluation and Magnetic Resonance Imaging

There is a relation between presence and quantity of MRI deviations (Tinelli et al., 2020) and visual functioning in children with a risk of CVI. Considerable damage, however, may be found in children with adequate visual functions.

In children with deviations in visual functions that have been referred, a relation seems to be found between MRI abnormalities (global atrophy/ischemic encephalopathy or structural malformations, agenesis of corpus callosum, porencephalic cysts, lissencephaly, Chiari malformation) and the presence of CVI.

Abnormalities of the MRI especially of the visual system can support the diagnosis CVI and can give more insight in the etiology of CVI. A normal MRI, however, does not exclude the diagnosis CVI. If making an MRI is considered this can be accompanied by a pediatric neurological examination.

Sometimes general anesthesia is necessary for successful MRI. However, this is invasive and implies an increased risk for the child. Because of these reasons the authors advise to be reluctant with indications for MRI.

Alternatives for anesthesia can be considered (McGuirt, 2016). In young children (<1 year) ultrasound of the brains can be considered.

*In situations where there is a strong clinical suspicion for CVI the authors recommend to consider MRI. Other causes of CVI, where therapy it needed, such as hydrocephalus, hypoglycemic damage, intracranial pressure on chiasm or retro chiasmal, for instance by a tumor, should be excluded by MRI before rehabilitation is started.*

### Topic Five: Genetic Assessment

The authors suggest a case-to-case approach in which the clinical geneticist uses an actual literature search on the genetic disorder to decide whether screening on CVI in the specific patient is justified. If CVI has been described several times or even just once if only a few patients are known with the disease, we advise referral to an ophthalmologist for structural examination on CVI.

In a subset of children with CVI, there is no plausible cause (Bosch et al., 2014b). These children need to be referred to a clinical geneticist. Genetic examination of a small group of patients (*n* = 56) with CVI without a plausible cause resulted in an explanation in 30–50% of cases (Bosch, 2015). Therefore, the authors recommend that children with CVI without a clear explanation for the CVI in their history and/or MRI of the brain, should be referred to a clinical geneticist. Except if genetic examination was performed in the last 3–5 years.

*The clinical geneticist can discuss advantages and disadvantages of genetic assessments. There can be several reasons to perform genetic testing.*

- *To answer the “why” question.*
- *Is there a recurrence risk (for the child, parents, or other family member) are co-morbidities to be expected and is screening or treatment indicated?*
- *More information on the developmental possibilities and future perspectives.*
- *Parents can contact other parents with a child with the same disorder.*

Based on the recent data a genetic diagnosis alone has a limited role in the diagnosis of CVI. The genetic diagnosis is at most useful to support the diagnosis CVI.

Besides ophthalmological and orthoptic examination, performed in specialized centers other assessments can be planned. Multi-disciplinary cooperation is necessary to organize this, if necessary, between different centers.

When a genetic diagnosis is made, the geneticist can consider to refer to an ophthalmologist for examination for CVI if:

Cerebral visual impairment is described several times in literature for this diagnosis.
Cerebral visual impairment is described as least once in literature and only few persons are known with this genetic disorder.

Discuss the outcome of the genetic examinations in a multidisciplinary team and use the findings in diagnostic follow up.

## Capacity and Working Load

As a result of the recommendations mentioned, the availability of financial means and health care professionals as well as the applicability and necessity of measures play an important role.

It is not always possible for diagnostic centers to organize an ophthalmologist and orthoptist and neuropsychologist with specific knowledge about CVI. Further training and schooling are necessary. Furthermore in a peripheral hospital setting and academic medical center visual fields are not often measured routinely and eye tracking or OCT are not performed regularly in children either.

Neonatal encephalopathy (NE) is the clinical manifestation of disordered neonatal brain function (Kurinczuk et al., 2010). Lack of universal agreed definitions of NE and the sub-group with hypoxic Ischemic Encephalopathy (HIE) makes the estimation of incidence and the identification of risk factors problematic. NE incidence is estimated as 3.0 per 1,000 live births (95%CI 2.7 to 3.3) and for HIE is 1.5 (95% CI 1.3 to 1.7), 60% of the children with severe Hypoxic Ischaemic Injury have CVI (Good et al., 2001; Fazzi et al., 2007; Solebo et al., 2017) and a large group of children with congenital abnormalities of brain have CVI as well. This means that in the least this group has to be examined for CVI by ophthalmologists and orthoptists. In several countries the incidence of VI and CVI has been investigated (Riise et al., 1992; Rosenberg et al., 1996; Rahi and Cable, 2003).

The number of children that can actually been seen by an ophthalmologist, is dependent on the capacity of ophthalmologists. This may be different in different countries. However, this diagnostic work can be better performed by a medical specialist. It is also preferable that the whole diagnostic process is supervised by a medical specialist as there are too many factors involved that may influence the future of the child and likewise the risks of missing a progressive disease in this diagnostic process.

## Data Availability Statement

The original contributions presented in this study are included in the article/Supplementary Material, further inquiries can be directed to the corresponding author.

## Author Contributions

All authors listed have made a substantial, direct, and intellectual contribution to the work, and approved it for publication.

## Conflict of Interest

The authors declare that the research was conducted in the absence of any commercial or financial relationships that could be construed as a potential conflict of interest.

## Publisher’s Note

All claims expressed in this article are solely those of the authors and do not necessarily represent those of their affiliated organizations, or those of the publisher, the editors and the reviewers. Any product that may be evaluated in this article, or claim that may be made by its manufacturer, is not guaranteed or endorsed by the publisher.

## Footnotes

1
http://www.gradeworkinggroup.org/

2
https://www.embase.com
