# Supplementary material for: The Multidisciplinary Guidelines for Diagnosis and Referral in Cerebral Visual Impairment
Source: Front Hum Neurosci. 2022 Jun 30;16:727565. doi: 10.3389/fnhum.2022.727565 (PMC9280621; doi:10.3389/fnhum.2022.727565)
Supplement: Supplementary file 1 [file Data_Sheet_1.pdf]

Appendix: Tables & Figures

Table 1A Evidence-table question 1A Topic one: Medical history and CVI ....
Table 1B Evidence-table question 1B Topic One: Questionnaires and CVI ....
Table 2A Evidence table question 2A Topic two: Ophthalmological and orthoptic investigations in CVI general hospitals..
Table 2B Evidence table question 2B Topic two: Ophthalmological and orthoptic investigations in CVI academic centers..
Table 3 Evidence-table question 3 Topic three: Neuropsychological assessment ....
Table 4 Evidence-table question 4 Topic four: Neuroradiological evaluation and MRI ....
Table 5 Evidence-question 5A Topic five: Genetic assessment ....
Table 6 Quality assessment for systematic reviews of diagnostic studies ....
Figure A Search string Topic one: Medical history and CVI ....
Figure B Search String Topic two: Ophthalmological and orthoptic investigations in CVI ....
Figure C Search string Topic three: Neuropsychological assessment..
Figure D Search String Topic four: Neuroradiological evaluation and MRI ....
Figure E Search String Topic five: Genetic assessment..

Tables

Table 1A Evidence-table question 1A Topic one: Medical history and CVI

Question 1A: Which elements does the clinical history need to contain in order to screen for CVI?

Evidence table for intervention studies (randomized controlled trials and non-randomized observational studies (cohort studies, case-control studies, case series))1

This table is also suitable for diagnostic studies (screening studies) that compare the effectiveness of two or more tests. This only applies if the test is included as part of a test-and-treat strategy – otherwise the evidence table for studies of diagnostic test accuracy should be used.

**Study reference**	**Study characteristics**	**Patient characteristics [2]**	**Intervention (I)**	**Comparison / control I [3]**	**Follow-up**	**Outcome measures and effect size [4]**	**Comments**
Wong, 1991	Cohort study	patients with severe visual loss in the presence of normal pupillary response and normal fundi, admitted between 1985 and 1990.<br><br>N: 34<br>age ± SD:<br>1 month to	Multidisciplinary evaluation, Flash VEP, EEG, photic stimulation, cranial CT	NA	1-5 years	Congenital Cortical Blindness: 7<br>Lissencephaly 3<br>Mitochondrial myopathy 1<br>Cerebral palsy 4<br>Abnormal VEP: 6<br>Abnormal EEG: 7<br>Acquired cortical blindness: 27<br>Hypoxia: 14<br>Severe perinatal asphyxia: 1<br>Cardiac arrest: 2<br>Severe hypotensive episodes following cardiac surgery: 5<br>Status epilepticus: 6<br>Encephalitis or meningitis: 6	-

		14 years<br>Sex:<br>59% M				Hydrocephalus with shunt malfunction: 1<br>Hydrocephalus with subdural hematoma, cerebral thrombosis, intracranial hemorrhage, and head trauma in 1 each<br>Unclear: 3<br>Cerebral palsy: 19<br>Mental retardation: 22<br>Myoclonic epilepsy: 7<br>Generalized tonic-clonic epilepsy: 6<br>Complex partial seizure: 1<br>Deafness: 1<br>VEP: 6/23 normal<br>EEG: 5/24 normal<br>Cranial CT: 11/27 cerebral atrophy<br>Bioccipital infarction: 1	
Chen, 1992	Cohort study Record review	CVI diagnosis N: 30 infants Age range: 6 - 12 months Sex: 43% M	Describe intervention (treatment/procedure/test):	Describe control (treatment /procedure /test):<br>-	Minimum 12 months	Devel delay: 73%<br>Seizures 57%<br>CP 33% premature 30%<br>hearingdef.17%<br>microcephaly7%<br>hydrocephalus30%<br>hypoxia 27%<br>IC hemorrhage 23%<br>meningitis 17%<br>Nystagmus (30%) Widening of the palpebral fissure (60%) Exotropia (50%) 15 esotropia (10%) Common refractive error- hyperopia<br>Abnormality in the visual pathway (68%)	CVI was diagnosed if the infant had poor vision, absent optokinetic nystagmus, normal pupillary reflexes, and normal ocular structure
Houliston, 1999	Cohort study	C=200 NS 5-12 years N=52 with hydrocephalus selected: binocular visual acuity at least 6/60, 5 years of age or older, Age range: 5-12 years, N: 52, Sex: 56%M	Standardized clinical history taking	Clinical history taking to children without cognitive disorders	NA	27 with cognitive problems, 16 out of 27 have problems with: shape recognition, simultaneous perception, perception of movement, colour perception, orientation, object recognition, and face recognition	Patiens with hydrocephalus Partly developmental questionnaire
Huo, 1999	Retrospective	CVI	NA	NA	Average	170 patients with	CVI -vision

	study	diagnosis<br>N: 170<br>Avg age: 3years			length: 5.9 years<br>Loss-to-follow-up: 10%<br>Reasons (describe): 56.5% returned for further evaluation<br>.	CVI were 2.4% of all patients seen 38 patients 22.4% had homonymous hemianopsia.<br>Perinatal hypoxia 38 (22.4%)<br>CVA 14.1%<br>Meningitis/encephalitis 12.4%<br>Acquired Hypoxia10%<br>Hydrocephalus9.4%<br>Idiopathic9.4%<br>Premature (<34 weeks)7.7%<br>Intracranial cyst5.3% Head trauma 4.1%<br>Seizures 4.1%<br>Congenital Microcephaly2.9%<br>Brain tumor2.4% In utero drug exposure 1.8%Other 6.5%	loss in the absence of signs of anterior visual pathway disease.<br>Vision loss greatly exceeding that which would be expected, given the findings of an ocular examination.
Shah, 2006	Retrospective cohort study	Cortical blindness<br>N: 345<br>Sex:<br>59% M<br>Mean age: 30 months (18-36 months)<br>Important prognostic factors:<br>1 Apgar score of less than 5 at 5 minutes,<br>2 need for mechanical ventilation immediately after birth<br>3 moderate to severe encephalopathy	logistic regression model of only 3 predictors with high statistical significance (administration of chest compressions >1 minute, onset of breathing >30 minutes, and base deficit value >16 in any blood gas analysis within the first 4 hours from birth)	NA	24 months	115 died, 111 had severe adverse outcome and 119 did not have this outcome<br><br>severe adverse:death or severe disability<br>Six independent predictors identified. Using the 3 most significant predictors (chest compressions, age at onset of respiration, and base deficit), severe adverse outcome rates were 46% (95% confidence interval, 33%-58%) with none of the 3 predictors, 64% (95% confidence interval, 54%-73%) with any 1 predictor, 76% (95% confidence interval, 66%-85%) with any 2 predictors, and 93% (95% confidence interval, 81%-99%) with all of the 3 predictors present.	Six independent predictors of outcomes were identified.<br>data are not separately presented for cortical blindness
Khetpal, 2007	Patient series<br>Chart review	CVI diagnosis<br>N: 98	Clinical picture<br><br>Ophthalmologic evaluation	NA	0.5 -10 years<br>The visual function at	C Etiology: perinatal hypoxia (35%) prematurity (29%), hydrocephalus	Most improvement in CVI- better initial visual acuity- good

			Neurologic evaluation<br><br>Ocular evaluation:<br><br>Visual acuity:<br><br>Neuroimaging with MRI or CT		final follow-up was compared with initial examinati on	(19%), central nervous system abnormalities (11%), seizures (10%).<br>had multiple etiologies (69%)<br>Ophthalmologic abnormalities: esotropia (19%), exotropia (40%), nystagmus (21%), and optic atrophy (42%).<br>neurological findings seizures (60%), cerebral palsy (37%), periventricular leukomalacia (12%), hemiparesis (21%), and hearing loss (11%).<br>Ocular abnormalities: Strabismus was common and was typically exotropic. Significant refractive error (>+3.00 D or <-2.00 D) was common (20%).<br>Visual acuity: Visual acuity tended to improve slightly over<br>time, although 40% had no improvement<br>MRI or CT: in 70% abnormal in 83%	prognosis
Ortibus, 2009	Observational study	Preterm<br>Age range: 4-20 years<br>Mean age ± SD: 7.0 ± 3<br>N: 35 (male) 35 (female)	L94 test battery consisting of five tests.	NA	NA	One-third had diagnosis visual perceptual deficit.<br>In CVI about half of the patients had a history of preterm birth.<br>ranking as follows: <Pc 5=1 (worst result); Pc 5 to 10=2; Pc 10 to 25=3; Pc 25 to 50=4; Pc 50 to 75=5; >Pc 75=6 (best result).<br>different subtasks distribution above or under Pc 50: VISM is the only subtask scored >Pc 50 in most of the patients (n=55 or 78%). OVERL, NOISE, DE VOS,	

						and VIEW are nearly equally distributed. Subtask profiles showed considerable variability. VIEW was situated in the £Pc 5 category in 24%. variability in subtask profiles different visual perceptual abilities. Preterm children birth and a clinical CP picture are at risk for a specific Perceptual visual impairment (PVI)	
Van Genderen, 2012	Cross-sectional study	N total at baseline: CVI N=30 NS N=23 CVI diagnosis Mean age 8 year Sex: I: % M C: % M			NA	CVI: 21 prematurely , < 35 weeks of gestation, 7 suffered perinatal asphyxia, and 2 developmental brain abnormality. 20 cerebral palsy, 8 children had epilepsy. 86% MRI abnormalities premature: periventricular leukomalacia; perinatal hypoxic-ischemic events in term-born cerebral infarction. MRI normal in 4 children,. optotype acuity of more than 20/40 (less than 0.3logMAR), 6 visual acuity of 20/20 (0.0logMAR); 41% (12/29) had a crowding ratio >=2.0. Visual field defects were found in 53% (16/30) of children. partial optic atrophy in 5 children significant refractive error in 3 (2 hyperopia, 1 myopia); 10 children had nystagmus. The CVI questionnaire showed 3 or more affirmative answers in all children; dorsal stream problems were reported more frequently than	In children without CVI only 23% had an abnormal clinical history. Only 1 MRI out of 6 was abnormal.

						ventral problems.	
Mac-Intyre-Beon, 2013	Prospective case control study of a cohort of prematurely born children	Preterm N-Intervention: 46<br>Control: 130<br>Age range: 5.5-12.3 years<br>Sex:<br>I: 63% M<br>C: % M	Ophthalmic, IQ, visual perception and structured clinical question inventory		NA	Visual perception tests (visual closure, global form, global motion, face processing) , prematurely born children had poorer scores than controls 15 (33%)of the prematurely born children had visual difficulties prematurely born had abnormalities of contrast sensitivity, and eye alignment and stereoacuity, but such differences were not identified for measured visual fields or visual acuity. The feature common to all the cases is difficulty handling complex visual scenes. Premature group performed worse than the controls in all tests	Prevalence of CVD in preterm: 21-47%
Bosch, 2014	Cross-sectional study Etiology	Diagnosis of CVI and low vision<br>N: 309<br>Sex: 55 % M<br>Age first examination: 36 (4 months-45 years)<br>Age most recent examination: 53 (5 months-45 years)	Genetic causes and brain abn (MRI)/ visual functions and ophth assessments	NA	NA	putative cause for CVI in 60% (184/309) of the cohort. In 40% etiology could not be determined<br>Severe perinatal problems (23%)<br>Exogenous factors (11%)<br>Hydrocephalus (6%)<br>West syndrome (9%) Genetic diagnosis (21%)	CVI can be part of a genetic syndrome and that abnormal ocular findings are present more frequently in acquired forms of CVI.
Geldof, 2015	Comparative, cross-sectional study	NInterv.= 105<br>Control: 67<br>Sex: 51% MVP/VLBW<br>Age: 5.5 y corrected age; very preterm birth (<32 wk. of gestation) /very low birth weight (birth weight	Describe intervention (treatment/procedure/test):<br>Very preterm birth	Describe control (treatment /procedure /test):<br>Controls were recruited from regular schools	NA	VP/VLBW children with CVI had lower performance IQ, but not verbal IQ, than those without CVI. 25 (24%) of the VP/VLBW children met criteria for CVI, compared to 5 (7%) of controls (P = 0.006, OR: 3.86, 95% CI: 1.40–10.70).	CVI was defined after comprehensive oculomotor, visual sensory and perceptive assessment, and validated against vision problems in daily life<br>Term children with CVI were excluded from further analysis.

		<1,500 g; VP/VLBW					
Ozturk, 2016	Cross-sectional, retrospective chart review	N total at baseline: 695<br>BCVA less than 0.3 in their better eye using the Snellen chart<br><br>Important prognostic factors2:<br>Mean age ± SD: 47.0 ± 51.9 months<br><br>Sex:<br>58 % M<br>diagnosis<br>Mean age: 47.0±51.9 months<br>N: 695<br>Sex:<br>58 % M	NA	NA	NA	CVI was present in 212 cases (30.5%) and 20.3% had premature birth. risk factors for CVI in our population: Asphyxia, metabolic disorders, genetic syndromes, infections and trauma.<br>The time of insult was unknown in 278 patients (40.0%), of whom 61.5% had CVI, including hypoxic ischemic encephalopathy (HIE), cerebral palsy, neurometabolic disorders and hydrocephalus. Chorioretinal dysplasia (21.6%) and primary optic atrophy (9.0%) were the other common pathologies in such cases.	Bilateral decreased visual response caused by damage to either the visual cortex or the geniculostriate visual pathways based on MRI findings was defined as cortical visual impairment (CVI), unless any ocular abnormalities related to visual loss existed.
Henderson 2017	Cross-sectional Diagnostic test	N=105 pos serology Zika, included N=70 mean age 16 weeks	MRI and CT-scans			70 of 74 (95%) occipital volume loss, 9 (12%) optic nerve atrophy, 3 (4%) chiasmal atrophy 1 (1%) showed an ocular calcification,<br>A total of 34 (55%) structural ocular abnormality 26 (42%) ocular abnormality both eyes. available visual acuity data, all had visual impairment	
Ventura 2021	Cross-sectional Diagnostic	Incl children with microcephaly and pos Zika serology N=32 mean age 5.7 months	Neurologic assessment and MRI/ visual functions		6 months	Visual impairment 32 infants (100%). Retinal and/or optic nerve findings 14 patients (44%). All patients (100%) demonstrated neurological and neuroimaging abnormalities; 3 (9%) presented with late-onset of microcephaly.	

Notes:

1. prognostic balance between treatment groups is usually guaranteed in randomized studies, but non-randomized (observational) studies require matching of patients between treatment groups (case-control studies) or multivariate

adjustment for prognostic factors (confounders) (cohort studies); the evidence table should contain sufficient details on these procedures.

2. provide data per treatment group on the most important prognostic factors ((potential) confounders);
3. for case-control studies, provide sufficient detail on the procedure used to match cases and controls.
4. for cohort studies, provide sufficient detail on the (multivariate) analyses used to adjust for (potential) confounders.

Table 1B Evidence-table question 1B Topic One: Medical history and CVI
Question 1B: Which questionnaire should be used in case the professional has a suspicion of CVI, based on the clinical history?

**Study reference**	**Study characteristics**	**Patient characteristics**	**Index test (test of interest)**	**Reference test**	**Follow-up**	**Outcome measures and effect size**	**Comments**
Ortibus, 2011	Validation study	CVI<br>N= 91<br>Age at examination (months): 82 (41-204)<br>Sex: 64% M /36 % F	CVI questionnaire of 46 closed items on six domains: Evaluating Visual Attitude, Ventral and dorsal stream functions, Complex (visuomotor) abilities, use of other Senses and associated CVI characteristics	Neuroophthalmological evaluation, Cognitive assessment, Visual perceptual assessment		Sum score based on 6 domains and clinical test score (L94) resulted in AUC of 0.69	CVI questionnaire
MacIntyre- Beon, 2012	Validation study	CVI diagnosis<br>N-<br>Patients: 36<br>Controls: 156<br>Mean age ± SD:<br>Patients: 10.8 ± 3.1<br>Controls: 8.2 ± 1.9	51-question inventory, in seven sections.	51-question inventory, in seven sections.	NA	The intra-class correlation was 0.98.	Validation study described in a letter to the editor, without a reference test
García-Ormaechea, 2014	Validation study	CVI diagnosis<br>N=220<br>Mean age (months) ± SD:<br>NVM: 10.6 ± 6.8)<br>AVM: 11.2 ± 7.2<br>Sex:<br>NVM: 53.5% M<br>56.5% M	Preverbal Visual assessment (PreVias) Questionnaire	Clinical behavioral assessments for the same four domains	Time between the index test and reference test	*specificity*<br>visual attention 86.5% visual communication 89.5% visual–motorcoordination 81.5% visualprocessing 81.3%<br>*sensitivity*<br>visual attention 79.2% visual communication 64.2% visual–motor coordination 77.9% visual processing 67.5%	Focuses on abnormal visual maturation Gives indication for further assessment 24mnd.

						Specificity 81.5%-89.5% Sensitivity 67.5%-79.2% *Test–retest* reliability visual attention 0.94-0.98	
Philip, 2016	Validation study	N=342 CVI diagnosis Mean age ± SD: 3.8 (Range 0-17) Sex: 35.7% M / 64.3% F	Structured Clinical Question Inventory (SCQI)	NA	NA	Good face validity, content validity, construct validity: good internal consistency: 0.92 to 0.94.	Further empirical testing needed before using in other cultures
Salavati, 2017	Face validity study	N=82 CP with and without CVI Mean age ± CP: 11.5 ± 4 CVI: 10 ± 3.5	CVI-MQ for children with GMFCS levels I, II and III: motor items about higher motor skills walking, stairclimbing and jumping, while the CVI-MQ for children with GMFCS levels IV and V contains motor skills such as rolling over and reaching.	NA	NA	The results of the ROC curve for CVI-MQ, GMFCS levels I, II and III are sensitivity 1.00 and specificity 0.96 CVI-MQ, GMFCS levels IV and V sensitivity 0.97 and specificity 0.98.	two CVI-Motor Questionnaires for children with CP: one for GMFCS levels I, II and III and one for GMFCS levels IV and V.
Gorrie 2019	Diagnostic online survey	N=535 535 participants Age Range = 5.01–18.99; Child Sex = Male 60%: Female 40%) 63% mainstream School 15% attended a mainstream school with a classroom assistant 4% a special class within a ainstream school, 14%	CVI questionnaire (Ortibus) and 5 questions			children with neurodevelopmental disorders, a large proportion have parent-reported CVI (23%-39%) and potential CVI (6.59-22.53% good convergent validity, internal consistency and a reliable factor structure and may therefore be suitable as screening tools	Whether or not a child had a diagnosis of CVI and/or additional neurodevelopmental disorders was based on parental report.

		attended special school and 4% were home schooled					
Hellgren 2020	Prospective population based Diagnostic	N=120 <27 weeks' gestational age (66 males; mean, 25.4 ± 1.0 weeks) and 97 full-term controls (56 males; mean, 39.9 ± 1.1 weeks) Both groups at the age of 6.5 years,	EXPRESS questionnaire	Assessments; Visual, perceptual, cognitive		Parents EPT reported more CVI features than the parents of controls; median sum scores of 25 (95% CI, 18.1-31.9) and 11 (95% CI, 8.8-13.2), respectively (P < 0.001), and a median difference of 14 (95% CI, 6.6-21.4)	
Moon 2021	Diagnostic prospective	N=201 typically developing age range (age 32.4±20.1 months, mean±standard deviation). 6 age range groups	PQCVI 23 questions based on a modified version of Houliston and Dutton's questionnaire	No visual function assessments	NA	the CVF score and scores to ventral-stream and dorsal-stream visual functions plausibly increased with age. The scores rapidly reached 90% of their maximum values up to the age of 36 months	PQCVI items produced reliable responses in children younger than 72 months. No data available on PQCVI in CVI
Chandna 2021	Case control	CVI: n=33 age 7.4 ±2.8 controls n=111 age 8.7±2.8	HVFQ1 51	Visual function ssessments to obtain diagnosis CVI	NA	the HVFQI-51 can detect a range of Higher visual functions (HVFDs) in children with CVI with good visual acuity and clearly distinguishes these children from typically developing children.	The inventory was adapted from the CVI-I (Dutton et al., 2010b; Macintyre-Beon et al., 2012) modifications were made

Table 2A Evidence table question 2A Topic two: Ophthalmological and orthoptic investigations in CVI

Question 2A: Minimal investigations required in CVI by ophthalmologist and orthoptist in a general hospital.

Evidence table for intervention studies (randomized controlled trials and non-randomized observational studies (cohort studies, case-control studies, case series))1

This table is also suitable for diagnostic studies (screening studies) that compare the effectiveness of two or more tests. This only applies if the test is included as part of a test-and-treat strategy – otherwise the evidence table for studies of diagnostic test accuracy should be used.

**Study reference**	**Study characteristics**	**Patient characteristics [2]**	**Intervention (I)**	**Comparison / control (C) [3]**	**Follow-up**	**Outcome measures and effect size [4]**	**Comments**
Dutton,	Retrospecti	CVI	Ophthalmic	Visual	NA	90 of the 130	Incidence of

1996	ve clinical observationa	diagnosis<br>N: 130<br>Age range is from 0-16 years	and orthoptic asserssments	fields were assessed, particularly for evidence of homonymous hemianopia, in all children in whom behavioral or confrontation methods could be used.		patients referred showed evidence of visual dysfunction due to cortical visual impairment	deviant visual acuity and visual field in CVI
Fazzi, 2007	Cross sectional study	CVI diagnosis<br>N: 121<br>*Age (mean)* ± SD: 54 months (3-180m), SD 39.7m<br>Sex: 59% boys	Ophthalmic, orthoptic and neuropsychological assessments	NA	NA	Reduced visual acuity was found in 105 of 121 (87%)<br>reduced contrast sensitivity in (80%), abnormal optokinetic nystagmus in 73%, and visual field deficit in 65%. Fixation was altered in 58 absent in 20% unstable in 26%<br>smooth pursuit absent or discontinuous in 79% and saccadic movements absent or dysmetric in in 34%<br>Strabismus was present in 88 (73%) of patients.<br>Abnormal ocular movements : were found in 43 patients (36%).<br>Impaired Visual perceptual abilities:<br>In 22% patients	Deficit of visual function caused by damage to, or malfunctioning of, the retro geniculate visual pathways in the absence of any major ocular disease.
Fazzi, 2012	Cross-sectional study	CP N: 129 children (54 females, 75 males)<br>*Age (mean) ± SD: 4y6m, SD 3y5m; (3m – 15y)*<br>*Sex: 58%*	Neurological and ophthalmic evaluation	NA	NA	Visual dysfunction in diplegia: refractive errors (75%) strabismus (90%), abnormal saccadic movements (86%), and reduced visual acuity	-

						(82%) reduced contrast sensitivity in 57% Hemiplegia showed strabismus (71%) and refractive errors (88%); oculomotor involvement (59%) altered visual field (64%). severe neuroophthalmological profile, characterized by ocular abnormalities (98%), oculomotor dysfunction (100%), and reduced visual acuity (98%) *reduced contrast sensitivity in 90%*	
Ruberto, 2006	Cross-sectional, comparative study	CVI N: 24 Age (mean) ± SD: 7.28 ± 2.7 NS= 88 subjects of similar age (mean, 7.9-2.7 years).	Heidelberg Retinal Tomograph (HRT)-II		NA	a highly significant probability in cup-to-disc area ratio (P <0.01, both eyes), rim area (P <0.01, both eyes), cup shape measure (P <0.01, right eye; P <0.01, left eye), and mean RNFL thickness (P <0.01, right eye; P <0.01, left eye). A novel observation was temporal atrophy of the optic nerve head in CVI.	-
Saidkasimova, 2006	Retrospective observationa a case series	CVI N: 7 Age (mean) ± SD: 7.9 ± 2.9 Etiology: 3/7 were born under 35 weeks of gestational age and 3/7 had seizures	Ophthalmic and orthoptic tests	NA	NA	Inferior visual field defect(3) strabismus (4) hyperopia (2) Visual acuity was better than 20/30 in all children: MRI scan each case; focal periventricular white matter abnormalities were present in similar locations ranging from mild to severe. .	-
Van der Zee, 2017	Cross-	CVI N total at	Orthoptic evaluation	Typically developin	NA	Median grating	Differentiate between children

	sectional exploratory study	baseline: 107<br>Age (median) ± SD: range 3-12y<br>Sex: 58% boys<br><br>heterogeneous group was recruited in the Rotterdam area through primary schools, two rehabilitation centers, and the Rotterdam Eye Hospital		g school children (group 1)		acuity only differed significantly between group 1 and 2 (tw(16) = 2.43,p = 0.03), whereas the differences in median uncrowded and crowded acuities between groups were all significant(p ≤ 0.02).	at risk of cerebral visual impairment and ocular visual impairment
Portengen 2020	Cross Sectional Patient group	CVI (n=115)	Visual field measurement (Full field peritest, Goldmann, and Behavioral)	no	no	Full field peritest had best reliability	
Mayer 2020	Unsel patient group	CVI n=20<br>ocularVI n=23	Contrast measurement	no	no	Good inter examiner reliability, contrast reduced in CVI	
Barsingerhorn 2018	Cross sectional case control	NS (n=94)<br>VI (n=30)<br>CVI (n= 17)	Symbol discrimination speed	no	no	Compared with NV, the intercept of the regression line was 170 6 28 ms higher for the children with VIo (t129 ¼ 6.16, P < 0.001), and 232 6 36 ms higher for the children with CVI (t129¼6.49, P < 0.001).	
Barsingerhorn 2019	Cross sectional case control	NS 88<br>VI 19<br>CVI 15	TAC computerized preferential looking test and eyetracking	no	no	The results revealed a significant effect of group (F(1, 65) = 16.26, p < 0.001): the saccade latencies of the children with visual impairment were on average 62 15 ms longer.	if relative fixation time (RTF) exceeded 50%<br>This method resulted in a total of 6/56 children on the finest grating, 3/56 children on the middle grating and 4/56 children on the coarsest

							grating who did not reach beyond 62.5% correct. The median accuracy with this method was 100%
Jacobsen 2019	Patient group	CVI (n=6) with Hemianopia cong. 3/acquired3	OCT ganglion cell layer thinning due to RTSD and fiber tractography of optic radiation	no	no	OCT findings (ganglion cell thinning, macula)corresponded with primary brain damage due to RTSD	
Luckman 2020	Cross sectional Patient group	CVI after arterial stroke (n=26)	Ophthalmol invest and VF after stroke	no	12 months for n=9	Vf def 7.7% blind (3.8%)	
Tinelli 2020	Diagnostic	CVI (PVL) N=72 mean gest. 32.4 weeks<br>Age at MRI 5.8 yrs	MRI			semi-quantitative MRI-scale for children with CP. lesion severity strongly correlated with visual function total score (global MRI score p = .000; hemispheric score p=.001 and subcortical score p=.000). Visual acuity, visual field, stereopsis and colour were compromised when a cortical damage was present, while ocular motricity (fixation and saccades) were compromised in presence of subcortical brain damage.	visual acuity, visual field, stereopsis and colour were compromised when a cortical damage was present, while ocular motricity (and in particular fixation<br>and saccades) were compromised in presence of subcortical brain damage
Huurneman, 2012	SR and meta-analysis of 22 quantitative studies	**VI AND CVI A**: N=13<br>Age 5-14y<br>**B**: N=42<br>Age 2-9y	**A**: <u>Stimulus</u>: LH single/LH line at 3m<br>**B**: <u>Stimulus</u>: Single Sheridan Gardener/7-letter Sheridan Gardener at 6m. Interoptotype spacing: 50% Foveal/eccentric: foveal	NA	NA	In children with CVI, crowding ratios were elevated.	In this review:<br>4) children with NV, 2) visually impaired (VI) children and adults and 3) children with cerebral visual impairment

							(CVI); this summary focuses on children with CVI.
Khetpal, 2012	Longitudinal study	CVI N: 98<br>Age (mean): 3.1 Y (0.2-19 y)<br>Sex: 57% M	Pediatric ophthalmologic examination	NA	161 patients with a diagnosis of CVI between 2002 and 2005 were identified, and 98 charts of patients were available for review	Visual function-poor - 75%, (LP)<br><br>40% had no improvement.<br>Strabismus was common and was typically exotropic 41%<br>Mild optic atrophy 25%<br>Nystagmus 21%<br>Refractive error <- 2.0 or >+ 3.0<br>Esotropia 19%<br>Moderate/severe optic atrophy 17%<br>Amblyopia 12%<br>Photophobia 4%<br>Retinal diseases 4%	

Notes:
1. prognostic balance between treatment groups is usually guaranteed in randomized studies, but non-randomized (observational) studies require matching of patients between treatment groups (case-control studies) or multivariate adjustment for prognostic factors (confounders) (cohort studies); the evidence table should contain sufficient details on these procedures;
2. provide data per treatment group on the most important prognostic factors ((potential) confounders);
3. for case-control studies, provide sufficient detail on the procedure used to match cases and controls;
4. for cohort studies, provide sufficient detail on the (multivariate) analyses used to adjust for (potential) confounders.

Table 2B Evidence table question 2B Topic two: Ophthalmological and orthoptic investigations in CVI

Question 2B: The more specialized diagnostics required in CVI by ophthalmologist and orthoptist in academic centers or diagnostic and academic centers.

Evidence table for systematic review of RCTs and observational studies (intervention studies)

Evidence table for intervention studies (randomized controlled trials and non-randomized observational studies (cohort studies, case-control studies, case series))[1]. This table is also suitable for diagnostic studies (screening studies) that compare the effectiveness of two or more tests. This only applies if the test is included as part of a test-and-treat strategy – otherwise the evidence table for studies of diagnostic test accuracy should be used.

**Study reference**	**Study characteristics**	**Patient 15haracteristic [2]**	**Intervention (I)**	**Comparison / control I [3]**	**Follow-up**	**Outcome measures and effect size [4]**	**Comments**
Cavascan, 2013	Retrospective cross-sectional	CVI diagnosis<br>N: 115<br>Age (median)	SWEEP VEP	NA	NA	VEP grating acuity deficit: GAD ranged from 0.17 to	To determine grating acuity

	observational study	± SD: 17 months, range 1.2 to 167 months<br>Sex: 57 % M				0.28 log units (mean 0.68 ± 0.27; median 0.71).	deficit magnitude measured by sweep-VEP in CVI
Clarke, 1997	Cohort study	CVI diagnosis<br>N: 44<br>Age not specified<br>Sex: not specified	Flash visual evoked potential	Visual response was assessed by a preferential looking technique fixation behavior, or response to a light source.	NA	NPV (84.6%)<br>PPV (45.1 %)<br>Sensitivity (87.5%)<br>Specificity (39.3%)	-
Frank, 1992	Case control	CVI diagnosis<br>N=60<br>Age ± SD: 25.7 months ± 32.7, range 6 weeks to 10 years<br>*Sex:*<br>*I: % M*<br>*C: % M*<br>*Cases were evaluated by age-matched controls.*	VEP pattern stimulation: consisting of 100 reversals of checks subtending 40’ of visual angle		NA	Every patient with cerebral blindness had abnormal VEPs.	Responses to flash and pattern stimulus from both primary and secondary visual cortical areas.
Good, 2012	Case control	CVI<br>N: 34<br>Age ± SD:<br>I: 1.94 ± 1.37 years<br>*Sex:*<br>*65 %M*<br>Controls N=16 age-matched healthy controls (mean +/- SD: 2.19 +/- 1.55).	sVEP measurements		NA	Reduced grating acuity (n:32)<br>Reduced contrast (30)	-to compare visual evoked potential measures of contrast sensitivity and grating acuity in children with CVI with those of age-matched typically developing controls.
							-
Kooiker, 2012	longitudinal study	CVI Age 1-12y<br>N I: 149<br>N C:127<br>Sex I: 60%<br>C: 50%	Eye tracking	Typically developing children recruited at daycare	In sufficient data for analysis -12	Risk group performed slower than control group Within the risk group, the prevalence of cerebral visual impairment, brain damage and intellectual disabilities was	-

						significantly higher in slow responding children compared to faster responding children. The presence of nystagmus	
Lim, 2005	Diagnostic Cohort	CVI N: 19<br>Cage: 6 months to 6 years.<br>History of birth at term, antenatal or perinatal distress or both, moderate to severe neonatal encephalopathy with seizures, and magnetic resonance imaging evidence of brain injury	VEP measurement:	Preferential looking acuity (PL) measurement	median duration of follow-up was 29 months (range, 24-76 months)	PL acuity: Nearly all visual acuities were below normal for age<br>VEP acuity: Improvement in VEP acuity was also on average 1 octave.	The rate of VEP acuity change and the final VEP acuity were not related to the neurological score
Sakai, 2003	Case series	CVI N: 9<br>Age 13.8 +/- 9.98<br>Sex: 78% male<br>subjects with severe motor and intellectual disabilities and CVI	(1) detection of optokinetic nystagmus<br>(2) detection of ocular pursuit			Most cases showed low contrast sensitivity-higher and lower spatial frequencies and high contrast sensitivity-middle	Diagnosis with CVI was made if five criteria were satisfied
Salati, 2002	Retrospective chart review	CVI/CP N: 51<br>Age (mean) ± SD: 7y 1m, ± 4y (range 2-16)<br>Sex: 66% males	Ophthalmological assessment:<br>Ocular motility assessment:<br>Visual scanning of the environment<br>Stability of fixation Quality of saccadic movements<br>Quality of smooth pursuit movements<br>Presence of paroxysmal ocular deviations<br>Variable-angle squint		NA	Paroxysmal l ocular deviations(78%); variable angle strabismus (86%); defective saccades (93%), fixation (88%)<br>Disorders of initiation and performing saccades, absence of smooth pursuit, vergence abnormalities, nystagmus beats, instability of fixation, and	-

						difficulty in the systematic exploration of the environment were observed	
Skoczenski, 2004	Case control study	CVI diagnosis<br>N: 35<br>Age (mean): 3y 6m (SD 3y 5m) (range 4m-16y)<br>Sex: 57% male	Grating acuity<br>Vernier acuity	Data from participants with normal vision are taken from Skoczenski and Norcia et al., 2002, and were collected with identical test parameters on the same apparatus.		Vernier and grating acuity were lower in patients with CVI compared with controls	-
Watson, 2007	Retrospective cohort	CVI diagnosis<br>N: 39<br>Age: 1 to 16 years, mean 5.0 SD 3.2 y<br>Sex:<br>I: % M<br>C: % M	Sweep VEP	NA	time between measures ranged from 0.6 to 13.7 years	The initial and final VA and contrast thresholds showed significant improvement Contrast threshold (available for 34 children): Mean initial Michelson contrast thresholds for the whole group was 7.03%, which represents substantial visual imp	
Watson, 2009	Case control study	CVI diagnosis<br>N: 37 (29)<br>Age range: 3.2 to 22.7 years<br>Sex:<br>I: % M<br>C: % M	Sweep VEP<br>Grating stimulus	Vernier Stimulus<br>PL Test	NA	The range of VEP grating acuity was from near normal (22.8 c/deg) to a deficit of nearly 0.8 log units (5.2 c/deg).	To determine the relationship between VEP vernier acuity, VEP grating acuity and behavioral grating acuity in patients with CVI
Watson, 2010	Retrospective cohort	CVI diagnosis<br>N: 33<br>Age ± SD: median age: 4.8y (range 1.3 to 19.2)	VEP technique	Behavioral acuity technique	average 6.9 years (SD 3.5 y)	average difference between the initial VEP acuity and the final behavioral	To determine if an early VEP measure of acuity is related to a

						acuity was 0.01 log (p=0.45).	young patient's future behavioral acuity
Howes 2021	Cohort Patient group	Retrospective, children with CVI seen in ophthalmology dep. (n=55)	PRVEP	no	no	VEP prediction of visual acuity	retrospective
Chang 2021	Cross-sectional	CVI 1-12yrs (n=16)	Eye tracking, grating acuity	Clinical VA measurement	no	Strong correlation between eye tracking VA and clinical Acuity	
Chandna 2021	Case-control	CVI with good VA CVI (n=31) NV (n=28)	Steady StateVEP of motion	Relative, rotary and absolute motion	no	Sign deficits in relative and rotary motion	
Raja 2021	Cross-sectional	CVI (n=218)	VEP	PLT (preferential looking)	Yes time..	VEP exceeded PLT by one or more octaves	retrospective
Tanke 2021	Case- control	Cvi (n=30) VI (n=33) NS (n=96)	Digital DEM	no	no	CVI,VI sign more time to read DEM than NS, CVI more than VI to read hor numbers/not vert	Relation between hor DEM and Crowding intensity
Kelly 2021	Cross sectional Patient group	CVI (n=30)	VEP, MRI, eye tracking, Diffusion tensor imaging tractography	no	no	Normal VEP and abnormal visual orienting behaviour	
Van der Zee 2019	Cross sectional	VI (n=38) VI + CVI (n=14) CVI (n=20)	Eye tracking	no	no	CVI and OCVI) performed significantly worse on the animate items than the group without brain damage (OVI).	Kaufman gestalt closure task
Pel 2016	Patient group	30 preterm children NS n=15	Reaction time to fixation on colour pattern and motion eye tracking	no	no	Longer response times in colour patternd and motion detection in pre-term born children	

Notes:

1. prognostic balance between treatment groups is usually guaranteed in randomized studies, but non-randomized (observational) studies require matching of patients between treatment groups (case-control studies) or multivariate adjustment for prognostic factors (confounders) (cohort studies); the evidence table should contain sufficient details on these procedures.
2. provide data per treatment group on the most important prognostic factors ((potential) confounders);
3. for case-control studies, provide sufficient detail on the procedure used to match cases and controls;
4. for cohort studies, provide sufficient detail on the (multivariate) analyses used to adjust for (potential) confounders.

Table 3 Evidence-table question: Topic three: Neuropsychological assessment
Question 3: Which tests can be used in neuropsychological assessment of CVI?

Evidence table for systematic reviews of diagnostic test accuracy studies

Study reference	Study characteristics	Patient characteristics	Index test (test of interest)	Reference test	Follow-up	Outcome measures and effect size	Comments
Auld, 2001	SR (and meta-analysis)	children with hemiplegia secondary to injury of the brain Details about age and gender- NA	Motor-free Visual Perceptual Test (MVPT) Test of Visual Perception Skills (TVPS) Developmental Test of Visual Perception (DTVP)	NA	NA	Test-retest reliability **MVPT- high** **TVPS-** high **DTVP** Not reported Internal consistency **MVPT**- high **TVPS-** from low to excellent **DTVP-** high	At present the DTVP and MVPT demonstrate the strongest clinometric properties and would and is recommended for clinical practice.
Stiers 2001	Patient control	N=79 Multiple disabled N=22 Prem with asphyxia N=57 age 2.75-6.50 years	L94			In 22 multiple disabled children (no indications of CVI)frequency of impairment did not exceed the reference sample for any L94 task In the 57 at risk for CVI a significant increase in the frequency of impairment was seen on six L94 tasks (range 12-38%).	
Van der Zee 2019	Cross sectional	VI (n=38) VI + CVI (n=14) CVI (n=20)	Eye tracking	no	no	CVI and OCVI) performed significantly worse on the animate items than the group without brain damage (OVI).	Kaufman gestalt closure task
Vancleef 2020 a	Diagnostic test Case-controls	Validation groups N=59 Normative sample N=301	Children's Visual Impairment test 3-6 year-old	-	-	**test-retest reliability (r=0.82, p<0.001, ICC=0.80) correlations with tests with a strong visual perception component (L94: r=0.74, p<0.001; SON-R 2.5-7: r=0.37, p=0.01) and low correlations with other tests**	**Lowest scores were observed for children with CVI compared to the other validation groups (F[3,44]=5.1, p=0.003).**

						**(Beery-VMI: r=0.25, p=0.09; SRS: r=0-0.26, p=0.09)**	
Itzhak 2021	diagnostic	Clinical records N=630 median age 77months	Consensus on visuo- perceptual dimensions Two questionnaires and Delphi (1) visual discrimination and matching, (2) object or picture recognition, (3) visual spatial perception, (4) figure-ground perception, (5) motion perception, (6) visual short-term memory, and (7) scene perception.	-		The most discriminating dimensions between CVI and no CVI were object/picture recognition (r = 0.56), visual spatial perception (r = 0.52), visual discrimination and matching (r = 0.47), and figure-ground perception (r = 0.39). Motion perception and visual short-term memory (both r = 0.22) were less discriminating	

Table 4 Evidence-table question Topic four: Neuroradiological evaluation and MRI

Evidence table for diagnostic test accuracy studies

**Study reference**	**Study characteristics**	**Patient characteristics**	**Index test (test of interest)**	**Reference test**	**Follow-up**	**Outcome measures and effect size**	**Comments**
Lambert, 1987	Case series, based on chart reviews. Retrospective.	CVI diagnosis N=75<br>Age:<br>Range: 0 – 6 years<br>Sex:<br>Not described.	CT or MRI scan	Visual acuity, counting fingers and light perception.	NA	diffuse atrophy (12), PVL (8) occipital lobe infarctions (6) watershed infarctions of the parieto-occipital region (2)<br>mild optic atrophy (6)	The visual recovery differed significantly with respect to the age at which the hypoxic insult occurred and CT and MRI
Uggetti, 1996	Case series based on chart reviews. Retrospective	CVI diagnosis N=27<br>Mean age ± SD:<br>34 months (range 16 months – 8 years)<br>Sex: 48% M / 52% F<br>Prematures, with cerebral palsy resulting from PVL.	MRI<br>.	Teller's visual Acuity Cards	NA	Cerebral visual impairment was observed in 17 (63%) subjects.	Cerebral visual impairment was defined as reduced sight despite normal ophthalmologic findings or findings too mild to explain the visual loss.
Sie, 2005	Longitudinal prospective study	CVI diagnosis N=53<br>Mean gestational age:	MRI	Binocular grating acuity	46 children underwent follow-up MRI at 18	Correlation- presence and extent of occipital white	With regard to visual impairment, the best predictor

		31.1 ± 5.6 weeks<br>Sex: 76% M / 24% F			months of age.	matter damage, as shown with neonatal and follow-up MRI and cerebral visual impairment..	was a serious abnormality of all three (general, motor and visual) neonatal MRI scores (sensitivity of 8/9 = 89%, specificity of 37/37=100%, positive predictive value of 8/8 = 100%, negative predictive value of 37 /38 = 97 %).
Khetpal, 2007	Case series, based on chart reviews. Retrospective.	CVI diagnosis<br>N= 98<br>Mean age ± SD:<br>3.1 years (range 0.2 – 19)<br>Sex: 57% M / 43% F	MRI and CT received at the time of diagnosis of CVI.	Visual function test	Time between the index test en reference test:<br>Unclear<br>Mean follow-up = 2,33 years.	Abnormal (83%)<br>Most patients; damage to both gray and white matter.<br>common - ischemic encephalopathy (42.2%)<br>periventricular leukomalacia (10.5%), and structural malformations (21%)<br>Optic atrophy (40%)	Children with HIE or hydrocephalus had greater improvement in visual function than those with other etiologies brain damage.
Van Genderen, 2012	Case series, based on chart reviews. Retrospective	CVI diagnosis<br>N=53<br>Mean age ± SD:<br>8 years (range 5-16)<br>Sex:<br>Not described	Index test MRI	Visual acuity, crowding ratio (CR), visual field assessment and results of ophthalmologic and orthoptic examination.	For how many participants were no complete outcome data available?<br>19N (36%)	All children with CVI, n=30, had an abnormal medical history.<br>CP (20),<br>21 were born prematurely before 35 weeks of gestation, 7 suffered perinatal asphyxia, and 2 had developmental brain abnormalities.<br>epilepsy (8)<br>(86%) of children had MRI abnormalities	
Philip 2020	review to evaluate the relationship between brain structure and CVI, as determined by MRI in		search of 5 database (PubMed, EMBASE, SCOPUS, CINAHL and Cochrane			MRI was found to have a strong association with CVI in all 30 studies. Only 13 (43 %) studies described	

	children with CP.		Database)			dorsal and/ ventral stream dysfunction	
Ho 2020	Diagnostic	CVI N=8	MRI	-	-	MRI: signs of cortical dysgenesis leading to congenital brain malformations such as polymicrogyria consistent with a prenatal timing of CNS injury.	Although subcortical white matter changes were common, signs of watershed injury to the visual cortex were absent, suggesting that the visual loss was attributable to a prenatal etiology with secondary birth complications.
Tinelli 2020	Diagnostic	CVI (PVL) N=72 mean gest. 32.4 weeks<br>Age at MRI 5.8 yrs	MRI			semi-quantitative MRI-scale for children with CP.<br>lesion severity strongly correlated with visual function total score (global MRI score p = .000; hemispheric score p=.001 and subcortical score p=.000).	visual acuity, visual field, stereopsis and colour were compromised when a cortical damage was present, while ocular motricity (and in particular fixation<br>and saccades) were compromised in presence of subcortical brain damage
Pamir 2021	Diagnostic, case control	CVI N= 12 17.36 years<br>Controls N=18 19.26 years	fMRI area V1 activation response profiles in V1; higher order (i.e. middle temporal cortex; area hMT+ stages of motion processing.	-	-	In V1, responses motion coherence were similar in both groups. In the CVI group, activation in area hMT+ was significantly reduced compared to controls<br>In CVI group: activation in area hMT+ was significantly reduced compared to controls, and consistent with a surround facilitation (rather than suppression) response profile.	

Table 5 Evidence-question 5 Topic five: Genetic assessment

Question 5: What is the position of genetic investigations in the diagnosis CVI?

Evidence table for intervention studies (randomized controlled trials and non-randomized observational studies (cohort studies, case-control studies, case series))1

This table is also suitable for diagnostic studies (screening studies) that compare the effectiveness of two or more tests. This only applies if the test is included as part of a test-and-treat strategy – otherwise the evidence table for studies of diagnostic test accuracy should be used.

**Study reference**	**Study characteristics**	**Patient characteristics [2]**	**Intervention (I)**	**Comparison / control (C) [3]**	**Follow-up**	**Outcome measures and effect size [4]**	**Comments**
Bosch, 2014a	Case control study	CVI diagnosis<br>N:<br>607, age:<br>1 patient born At term and a mean gestational age of: 38,7 weeks<br>Sex:<br>71 %M	chromosomal investigations.	The database of genomic variants (http://dgv.tcag.ca/dgv/app/<br>home) was used to filter against common variants and to compare with normal controls.	NA	46 chromosomal aberrations in 41 patients were associated with CVI	chromosomal aberrations are likely to be associated with CVI.
Bosch, 2014b	Observational study, retrospective, based on medical files	CVI, N:<br>309<br>*Sex:*<br>*Total CVI group:*<br>(170/309) 55% M<br>*Purely genetic:*<br>(17/28) 61% M	Medical files were investigated for genetic investigations to gain genetic data.	No comparison was made between the purely genetic group and the normal population.	NA	Patients (21%) a genetic diagnosis was obtained, of which 38 (12%) had other possible risk factors making differentiation between acquired and genetic not possible.	the outcome data of monogenetic cause are included
Bosch, 2016	Observational study	CVI diagnosis<br>N:<br>Intervention: 25<br>Control: not described<br>*Age, median (range):*<br>*I: 12 years (1-33)*<br>*C: not described*<br>*Sex:*<br>*I: 68% M*<br>*C: not described*	Whole-exome sequencing was used in patients and their parents to identify underlying genetic causes for CVI.	dbSNP allel frequency (<1%), inhuis frequency <1%.	NA	19 candidate CVI genes, and 11 genes unlikely to be related to CVI (20%)-genetic diagnosis for the CVI (44%) one or more candidate genes for CVI<br>.	
Wan 2019	review	NA				approximately 90%<br>of TSC patient have epilepsy, cortical brain malformations and/or neuropsychiatric disorders.	

						This implies soms degree of neurological dysfunction, which is highly correlated with CVI.	
Wilton 2021	Cross sectional and chart review	N=81 Children with Down syndrome Age 4,4-17 years 55,6% female	Online questionnair e	-	-	The mean total score for children with suspected CVI was 59.5% (SD = 10.5%, range = 41.2–78.8, Shapiro-Wilk $p > 0.05$) and the mean for children without suspected CVI was 44.1% (SD = 10.3%, range = 24.0–67.8, Shapiro-Wilk $p > 0.05$); the difference was significant ($t = 6.286$, $p < 0.001$).	Of the 81 children, 31 screened positive for suspected CVI; a prevalence of 38.3%. corrected visual acuity (binocular LogMAR), ametropia (best vision sphere of the least ametropic or fixing eye), magnitude of astigmatism (of least astigmatic or fixing eye), strabismus (present or not), accommodati on (accurate or not), nystagmus (present or not).

Table 6 Quality assessment for systematic reviews of diagnostic studies

Based on AMSTAR checklist (Shea, 2007, BMC Methodol 7: 10; doi:10.1186/1471-2288-7-10) and PRISMA checklist (Moher 2009, PLoS Med 6: e1000097; doi:10.1371/journal.pmed1000097)
Research question: Which tests can be used in neuropsychological assessment of CVI?

Study<br><br>First author, year	Appropriate and clearly focused question?[1]<br><br>Yes/no/ unclear	Comprehensive and systematic literature search?[2]<br><br>Yes/no/ unclear	Description of included and excluded studies?[3]<br><br>Yes/no /unclear	Description of relevant characteristics of included studies?[4]<br><br>Yes/no/ unclear	Assessment of scientific quality of included studies?[5]<br><br>Yes/no/ unclear	Enough similarities between studies to make combining them reasonable?[6]<br><br>Yes/no/ unclear	Potential risk of publication bias taken into account?[7]<br><br>Yes/no/ unclear	Potential conflicts of interest reported?[8]<br><br>Yes/no/ unclear
Auld, 2001	Yes	Yes	No	Yes, but not elaborate	no	Not applicable	Min 10 studies, not applicable	no
Huurneman 2012	Yes	Yes	Yes	Yes	Yes	yes	Yes sample size	No
Wan 2019	yes	no	no	yes	no	unclear	no	no
Philip 2020	Yes	Yes	Yes	Yes	Yes	Yes	Yes; CVI assessment and terminology	No

1. research question (PICO) and inclusion criteria should be appropriate (in relation to the research question to be answered in the clinical guideline) and predefined;
2. search period and strategy should be described; at least Medline searched;
3. potentially relevant studies that are excluded at final selection (after reading the full text) should be referenced with reasons;
4. characteristics of individual studies relevant to the research question (PICO) should be reported;
5. quality of individual studies should be assessed using a quality scoring tool or checklist (preferably QUADAS-2; COSMIN checklist for measuring instruments) and taken into account in the evidence synthesis;
6. clinical and statistical heterogeneity should be assessed; clinical: enough similarities in patient characteristics, diagnostic tests (strategy) to allow pooling? For pooled data: at least 5 studies available for pooling; assessment of statistical heterogeneity and, more importantly (see Note), assessment of the reasons for heterogeneity (if present)? Note: sensitivity and specificity depend on the situation in which the test is being used and the thresholds that have been set, and sensitivity and specificity are correlated; therefore, the use of heterogeneity statistics (p-values; I^2) is problematic, and rather than testing whether heterogeneity is present, heterogeneity should be assessed by eye-balling (degree of overlap of confidence intervals in Forest plot), and the reasons for heterogeneity should be examined;
7. there is no clear evidence for publication bias in diagnostic studies, and an ongoing discussion on which statistical method should be used. Tests to identify publication bias are likely to give false-positive results, among available tests, this test is most valid. Irrespective of the use of statistical methods, you may score “Yes” if the authors discuss the potential risk of publication bias;
8. sources of support (including commercial co-authorship) should be reported in both the systematic review and the included studies. Note: To get a “yes,” source of funding or support must be indicated for the systematic review AND for each of the included studies.
9.

Figures

Figure A Search string Topic one: Medical history and CVI

Database	Search terms	Total
Medline (OVID)<br><br>1946-mrt 2017 and mrt 2017-dec 2021<br><br>Engels	1 (cerebral visual impairment* or cortical visual impairment* or cognitive visual impairment*).ti,ab,kf. (242)<br>2 (cerebral vision impairment* or cortical vision impairment* or cognitive vision impairment*).ti,ab,kf. (5)<br>3 (cerebral visual disorder* or cortical visual disorder* or cognitive visual disorder*).ti,ab,kf. (8)<br>4 (CVI and (CP or "cerebral pals*" or vision or visual)).ti,ab,kf. (143)<br>5 ((brain adj3 ("visual impairment*" or "visual disorder*" or "vision impairment*")) or "cortical blindness" or "cerebral blindness").ti,ab,kf. (1141)<br>6 Blindness, Cortical/ (524)<br>7 ("Higher perceptual" adj2 (deficit* or dysfunction*)).ti,ab,kf. (1)<br>8 1 or 2 or 3 or 4 or 5 or 6 or 7 (1610)<br>15 Medical History Taking/ or "surveys and questionnaires"/ or vision screening/ or risk assessment/ or risk factors/ or Prognosis/ or History.ti,ab,kf. or (questionnaire* or screen*).ti,ab,kf. or (risk* or prognos* or anamnes*).ti,ab,kf. or ("query sheet*" or "inquiry form*").ti,ab,kf. or Diagnosis, Differential/ or (Differential adj2 diagnosis).ti,ab,kf. (4216394)<br>16 8 and 15 (420)<br>17 limit 16 to english language (363)<br>18 remove duplicates from 17 (353)<br>19 (meta-analysis/ or meta-analysis as topic/ or (meta adj analy$).tw. or ((systematic* or literature) adj2 review$1).tw. or (systematic adj overview$1).tw. or exp "Review Literature as Topic"/ or cochrane.ab. or cochrane.jw. or embase.ab. or medline.ab. or (psychlit or psyclit).ab. or (cinahl or cinhal).ab. or cancerlit.ab. or ((selection criteria or data extraction).ab. and "review"/)) not (Comment/ or Editorial/ or Letter/ or (animals/ not humans/)) (316448)<br>20 18 and 19 (15)<br>21 18 not 20 (338)	1007
Embase (Elsevier)	'cerebral blindness'/exp OR 'cerebral visual impairment*':ti,ab OR 'cortical visual impairment*':ti,ab OR 'cognitive visual impairment*':ti,ab OR 'cerebral vision impairment*':ti,ab OR 'cortical vision impairment*':ti,ab OR 'cognitive vision impairment*':ti,ab OR 'cerebral visual disorder*':ti,ab OR 'cortical visual disorder*':ti,ab OR 'cognitive visual disorder*':ti,ab OR (cvi:ti,ab AND (cp:ti,ab OR 'cerebral pals*':ti,ab OR vision:ti,ab OR visual:ti,ab)) OR (brain NEAR/3 ('visual impairment*' OR 'visual disorder*' OR 'vision impairment*')):ti,ab OR 'cortical blindness':ti,ab OR 'cerebral blindness':ti,ab OR ('higher perceptual' NEAR/2 (deficit* OR dysfunction*)):ti,ab<br><br>AND (english)/lim AND (embase)/lim<br><br>AND ('anamnesis'/exp OR 'questionnaire'/exp OR 'mass screening'/exp OR 'risk assessment'/exp OR 'risk factor'/exp OR 'prognosis'/exp/mj OR 'differential diagnosis'/exp OR questionnaire*:ti,ab OR screen*:ti,ab OR risk*:ti,ab OR prognos*:ti,ab OR anamnes*:ti,ab OR 'query sheet*':ti,ab OR 'inquiry form*':ti,ab OR (differential NEAR/2 diagnosis):ti,ab)<br><br>'meta analysis'/de OR cochrane:ab OR embase:ab OR psycinfo:ab OR cinahl:ab OR medline:ab OR (systematic NEAR/1 (review OR overview)):ab,ti OR (meta NEAR/1 analy*):ab,ti OR metaanalys*:ab,ti OR 'data extraction':ab OR cochrane:jt OR 'systematic review'/de NOT ('animal experiment'/exp OR 'animal model'/exp OR 'nonhuman'/exp NOT 'human'/exp)) (10)<br><br>Divers (466)	

Database	Search terms	Total
Medline (OVID)<br><br>1946-mrt 2017 and mrt 2017-dec 2021<br><br>Engels	1 (cerebral visual impairment* or cortical visual impairment* or cognitive visual impairment*).ti,ab,kf. (242)<br>2 (cerebral vision impairment* or cortical vision impairment* or cognitive vision impairment*).ti,ab,kf. (5)<br>3 (cerebral visual disorder* or cortical visual disorder* or cognitive visual disorder*).ti,ab,kf. (8)<br>4 (CVI and (CP or "cerebral pals*" or vision or visual)).ti,ab,kf. (143)<br>5 ((brain adj3 ("visual impairment*" or "visual disorder*" or "vision impairment*")) or "cortical blindness" or "cerebral blindness").ti,ab,kf. (1141)<br>6 Blindness, Cortical/ (524)<br>7 ("Higher perceptual" adj2 (deficit* or dysfunction*)).ti,ab,kf. (1)<br>8 1 or 2 or 3 or 4 or 5 or 6 or 7 (1610)<br>15 Medical History Taking/ or "surveys and questionnaires"/ or vision screening/ or risk assessment/ or risk factors/ or Prognosis/ or History.ti,ab,kf. or (questionnaire* or screen*).ti,ab,kf. or (risk* or prognos* or anamnes*).ti,ab,kf. or ("query sheet*" or "inquiry form*").ti,ab,kf. or	1007

	Diagnosis, Differential/ or (Differential adj2 diagnosis).ti,ab,kf. (4216394)<br>16 8 and 15 (420)<br>17 limit 16 to english language (363)<br>18 remove duplicates from 17 (353)<br>19 (meta-analysis/ or meta-analysis as topic/ or (meta adj analy$).tw. or ((systematic* or literature) adj2 review$1).tw. or (systematic adj overview$1).tw. or exp "Review Literature as Topic"/ or cochrane.ab. or cochrane.jw. or embase.ab. or medline.ab. or (psychlit or psyclit).ab. or (cinahl or cinhal).ab. or cancerlit.ab. or ((selection criteria or data extraction).ab. and "review"/)) not (Comment/ or Editorial/ or Letter/ or (animals/ not humans/)) (316448)<br>20 18 and 19 (15)<br>21 18 not 20 (338)	
Embase (Elsevier)	'cerebral blindness'/exp OR 'cerebral visual impairment*':ti,ab OR 'cortical visual impairment*':ti,ab OR 'cognitive visual impairment*':ti,ab OR 'cerebral vision impairment*':ti,ab OR 'cortical vision impairment*':ti,ab OR 'cognitive vision impairment*':ti,ab OR 'cerebral visual disorder*':ti,ab OR 'cortical visual disorder*':ti,ab OR 'cognitive visual disorder*':ti,ab OR (cvi:ti,ab AND (cp:ti,ab OR 'cerebral pals*':ti,ab OR vision:ti,ab OR visual:ti,ab)) OR (brain NEAR/3 ('visual impairment*' OR 'visual disorder*' OR 'vision impairment*')):ti,ab OR 'cortical blindness':ti,ab OR 'cerebral blindness':ti,ab OR ('higher perceptual' NEAR/2 (deficit* OR dysfunction*)):ti,ab<br><br>AND (english)/lim AND (embase)/lim<br><br>AND ('anamnesis'/exp OR 'questionnaire'/exp OR 'mass screening'/exp OR 'risk assessment'/exp OR 'risk factor'/exp OR 'prognosis'/exp/mj OR 'differential diagnosis'/exp OR questionnaire*:ti,ab OR screen*:ti,ab OR risk*:ti,ab OR prognos*:ti,ab OR anamnes*:ti,ab OR 'query sheet*':ti,ab OR 'inquiry form*':ti,ab OR (differential NEAR/2 diagnosis):ti,ab)<br><br>'meta-analysis'/de OR cochrane:ab OR embase:ab OR psycinfo:ab OR cinahl:ab OR medline:ab OR (systematic NEAR/1 (review OR overview)):ab,ti OR (meta NEAR/1 analy*):ab,ti OR metaanalys*:ab,ti OR 'data extraction':ab OR cochrane:jt OR 'systematic review'/de NOT ('animal experiment'/exp OR 'animal model'/exp OR 'nonhuman'/exp NOT 'human'/exp)) (10)<br><br>Divers (466)	

Figure B Search String Topic two: Ophthalmological and orthoptic investigations in CVI

**Database**	**Search terms**	**Total**
Medline (OVID) 1990-april 2017 and April 2017-dec 2021<br><br>Engels, Nederlands	1 (cerebral visual impairment* or cortical visual impairment* or cognitive visual impairment*).ti,ab,kf. (246)<br>2 (cerebral vision impairment* or cortical vision impairment* or cognitive vision impairment*).ti,ab,kf. (5)<br>3 (cerebral visual disorder* or cortical visual disorder* or cognitive visual disorder*).ti,ab,kf. (8)<br>4 (CVI and (CP or "cerebral pals*" or vision or visual)).ti,ab,kf. (147)<br>5 ((brain adj3 ("visual impairment*" or "visual disorder*" or "vision impairment*")) or "cortical blindness" or "cerebral blindness").ti,ab,kf. (1148)<br>6 Blindness, Cortical/ (528)<br>7 ("Higher perceptual" adj2 (deficit* or dysfunction*)).ti,ab,kf. (1)<br>8 1 or 2 or 3 or 4 or 5 or 6 or 7 (1626)<br>36 "Visual Acuity"/ or "Vision, Low"/di or "Visual Pathways"/ or "Visual Field Tests"/ or "Orthoptics"/mt or "Optic Atrophy"/ or "Vision, Binocular"/ or "Visual Perception"/ or exp Diagnostic Techniques, Ophthalmological/ or exp Ocular Motility Disorders/ or Diagnosis, Differential/ or (acuity or nystagmus or motility or crowding).ti,ab. or ((Visual adj (function* or field* or behavio?r* or pathway*)) or fixation or perimetry or refraction or stereopsis or "contrast sensitivity" or "colour vision" or fundoscopy or "visual evoked potential" or VEP or pupil*).ti,ab,kf. or (differentiating or identifying or characterising or "differential diagnosis" or "visual testing" or ((ophthalmological or ocular or orthoptic) adj2 findings)).ti,ab,kf. or diagnosis.ti. (1500097)<br>37 8 and 36 (671)<br>40 limit 37 to English language (601)<br>41 remove duplicates from 40 (583)	816

Figure C Search string Topic three: Neuropsychological assessment

**Database**	**Search terms**	**Total**
Medline (OVID)	2 (cerebral visual impairment* or cortical visual impairment* or cognitive visual impairment*).ti,ab,kf. (257)<br>3 (cerebral vision impairment* or cortical vision impairment* or cognitive vision impairment*).ti,ab,kf. (5)	716

	4 (cerebral visual disorder* or cortical visual disorder* or cognitive visual disorder*).ti,ab,kf. (8)<br>5 (CVI and (CP or vision or visual)).ti,ab,kf. (151)<br>6 ((brain adj3 ("visual impairment*" or "visual disorder*" or "vision impairment*")) or cortical blindness).ti,ab,kf. (1110)<br>7 Blindness, Cortical/ (539)<br>8 2 or 3 or 4 or 5 or 6 or 7 (1612)<br>9 (child* or schoolchild* or infan* or adolescen* or pediatri* or paediatr* or neonat* or boy or boys or boyhood or girl or girls or girlhood or youth or youths or baby or babies or toddler* or childhood or teen or teens or teenager* or newborn* or postneonat* or postnat* or puberty or preschool* or suckling* or picu or nicu or juvenile?).tw. or adolescent/ or exp child/ or exp infant/ or Pediatrics/ (3958537)<br>10 8 and 9 (656)<br>11 exp Neuropsychological Tests/ or ((neurocognitive or neurodevelopment* or neuropsycholog*) adj3 (assess* or test* or diagnos* or screen* or findings)).ti,ab,kf. (95877)<br>12 8 and 11 (48)<br>13 limit 12 to english language (43)<br><br>14 exp Vision Disorders/ or exp Visual Perception/ or exp Visual Acuity/ or exp Depth Perception/ or exp Perceptual Disorders/ or Visual Pathways/ or exp Pattern Recognition, Visual/ or ((visual adj2 cognit* adj (dysfunction* or impairment*)) or (visual adj cognit* adj (dysfunction* or impairment*)) or (visual adj percept* adj (dysfunction* or impairment*)) or (visual adj processing adj (dysfunction* or impairment*)) or (visual cognition or cortical vision or spatial vision or vis* development)).ti,ab. (282555)<br>15 exp Brain Diseases/cl, co, cn, pp or exp Cerebral Cortex/ or Corpus Callosum/ph or Leukomalacia, Periventricular/di, et, pp or White Matter/pa or exp Occipital Lobe/ph, pp or Temporal Lobe/ph, pp or Premature Birth/pp or Cerebral Palsy/co, pp or exp infant, very low birth weight/ or exp Genetic Predisposition to Disease/ or exp Chromosome Aberrations/ or exp Chromosome Mapping/ or Disabled Children/ or Developmental Disabilities/ or down syndrome/ or williams syndrome/ or DiGeorge Syndrome/ or Hypoxia-Ischemia, Brain/ or exp Optic Nerve Diseases/ or exp "Malformations of Cortical Development"/ or exp Hydrocephalus/ or exp Central Nervous System Infections/ or exp Epilepsy/ or exp Craniocerebral Trauma/ or exp Metabolic Diseases/ or exp Neurodegenerative Diseases/ or exp Chemically-Induced Disorders/ or Shaken Baby Syndrome/ or exp Hypoglycemia/co or "Pregnancy Complications, Infectious"/ or exp Cognition Disorders/ or Premature Birth/pa, pp (2857907)<br>16 ((Brain adj3 (Disease* or abnormalities)) or Cerebral Cortex or corpus Callosum or Occipital Lobe or "Periventricular Leukomalacia" or White Matter or Temporal Lobe or Premature Birth or "Very Low Birth Weight" or Cerebral Palsy or (Chromosome adj3 (Aberration* or mapping)) or Disabled children or Developmental disabilit* or Williams syndrome or Down syndrome or Velocardiofacial syndrome or hypoxic ischemic injur* or optic nerve hypoplasia* or cerebral malformation* or hydrocephalus or central nervous system infection or epilep* or head trauma* or metabolic diseases or neurodegenerative diseases or intoxication or poison* or Shaken baby syndrome or Hypoglycemic episode or Congenital infection* or Cognition Disorder*).ti,ab. (463915)<br>17 14 and (15 or 16) and 9 (19438)<br>18 exp Neuropsychological Tests/ or ((neurocognitive or neurodevelopment* or neuropsycholog*) adj3 (assess* or test* or diagnos* or screen* or findings)).ti,ab,kf. (95877)<br>19 17 and 18 (1367)<br>20 limit 19 to english language (1317)<br>21 20 not 12 (1306)<br>22 (meta-analysis/ or meta-analysis as topic/ or (meta adj analy$).tw. or ((systematic* or literature) adj2 review$1).tw. or (systematic adj overview$1).tw. or exp "Review Literature as Topic"/ or cochrane.ab. or cochrane.jw. or embase.ab. or medline.ab. or (psychlit or psyclit).ab. or (cinahl or cinhal).ab. or cancerlit.ab. or ((selection criteria or data extraction).ab. and "review"/)) not (Comment/ or Editorial/ or Letter/ or (animals/ not humans/)) (331922)<br>23 21 and 22 (14)<br>24 Epidemiologic studies/ or case control studies/ or exp cohort studies/ or Controlled Before-After Studies/ or Case control.tw. or (cohort adj (study or studies)).tw. or Cohort analy$.tw. or (Follow up adj (study or studies)).tw. or (observational adj (study or studies)).tw. or Longitudinal.tw. or Retrospective*.tw. or prospective*.tw. or consecutive*.tw. or Cross sectional.tw. or Cross-sectional studies/ or historically controlled study/ or interrupted time series analysis/ or comparative study.pt. (4297393)<br>25 21 and 24 (428)<br>26 25 not 23 (427)<br>27 remove duplicates from 26 (419)	

PsycINFO (OVID)	1 (cerebral visual impairment* or cortical visual impairment* or cognitive visual impairment*).ti,ab,kf. (108)<br>2 (cerebral vision impairment* or cortical vision impairment* or cognitive vision impairment*).ti,ab,kf. (3)<br>3 (cerebral visual disorder* or cortical visual disorder* or cognitive visual disorder*).ti,ab,kf. (7)<br>4 (CVI and (CP or "cerebral pals*" or vision or visual)).ti,ab,kf. (67)<br>5 ((brain adj3 ("visual impairment*" or "visual disorder*" or "vision impairment*")) or "cortical blindness" or "cerebral blindness").ti,ab,kf. (213)<br>6 Blindness, Cortical/ (0)<br>7 ("Higher perceptual" adj2 (deficit* or dysfunction*)).ti,ab,kf. (1)<br>8 1 or 2 or 3 or 4 or 5 or 6 or 7 (345)<br>9 exp Neuropsychological Tests/ or exp neuropsychological assessment/ (15826)<br>10 ((neurocognitive or neurodevelopmental or neuropsycholog*) adj3 (assess* or test* or diagnos* or screen* or findings)).ti,ab,kf. (21495)<br>11 Visual perception in preterm children: what are we currently measuring?.m_titl. (0)<br>12 9 or 10 (30545)<br>13 8 and 12 (5)<br>14 limit 13 to english language (2)<br><br>15 exp vision disorders/ (15357)<br>16 exp visual perception/ (81046)<br>17 exp depth perception/ (4072)<br>18 "pattern recognition (cognitive process)"/ (433)<br>19 ((visual adj2 cognit* adj (dysfunction* or impairment*)) or (visual adj cognit* adj (dysfunction* or impairment*)) or (visual adj percept* adj (dysfunction* or impairment*)) or (visual adj processing adj (dysfunction* or impairment*)) or (visual cognition or cortical vision or spatial vision or vis* development)).ti,ab. (1285)<br>20 15 or 16 or 17 or 18 or 19 (95727)<br>21 12 and 20 (855)<br>22 exp Neuropsychological Tests/ or exp neuropsychological assessment/ or ((neurocognitive or neurodevelopmental or neuropsycholog*) adj3 (assess* or test* or diagnos* or screen* or findings)).ti,ab,kf. (30545)<br>23 (child* or schoolchild* or infan* or adolescen* or pediatri* or paediatr* or neonat* or boy or boys or boyhood or girl or girls or girlhood or youth or youths or baby or babies or toddler* or childhood or teen or teens or teenager* or newborn* or postneonat* or postnat* or puberty or preschool* or suckling* or picu or nicu or juvenile?).tw. (910729)<br>24 20 and 22 and 23 (156)<br>25 limit 24 to english language (138)<br>26 limit 25 to "reviews (maximizes sensitivity)" (25)<br>27 limit 25 to ("0200 clinical case study" or "0300 clinical trial" or "0400 empirical study") (116)<br>28 27 not 26 (96)<br>31 26 not 14 (24)<br>32 27 not 14 (115)	

Figure D Search String Topic four: Neuroradiological evaluation and MRI

Database	Search terms	Total
Medline (OVID)<br><br>1946-juli 2017 and Juli 2017-dec 2021<br><br>Engels, Nederlands	2 (cerebral visual impairment* or cortical visual impairment* or cognitive visual impairment*).ti,ab,kf. (257)<br>3 (cerebral vision impairment* or cortical vision impairment* or cognitive vision impairment*).ti,ab,kf. (5)<br>4 (cerebral visual disorder* or cortical visual disorder* or cognitive visual disorder*).ti,ab,kf. (8)<br>5 (CVI and (CP or vision or visual)).ti,ab,kf. (150)<br>6 ((brain adj3 ("visual impairment*" or "visual disorder*" or "vision impairment*")) or cortical blindness).ti,ab,kf. (1110)<br>7 Blindness, Cortical/ (539)<br>8 2 or 3 or 4 or 5 or 6 or 7 (1611)<br>21 exp Magnetic Resonance Imaging/ or dg.fs. (1320707)<br>22 (neuroimaging or neuro-imaging or imaging or MRI* or tractograph* or HARDI or DWI or DTI or MR imag* or neuroradiology or fMRI or eyetracking).ti,ab,kf. (779778)<br>23 21 or 22 (1718646)<br>24 8 and 23 (635)<br>25 limit 24 to (dutch or english) (563)<br>26 remove duplicates from 25 (541)<br>27 (meta-analysis/ or meta-analysis as topic/ or (meta adj analy$).tw. or ((systematic* or literature) adj2 review$1).tw. or (systematic adj overview$1).tw. or exp "Review Literature as Topic"/ or cochrane.ab. or cochrane.jw. or embase.ab. or medline.ab. or (psychlit or psyclit).ab. or (cinahl or	723

	cinhal).ab. or cancerlit.ab. or ((selection criteria or data extraction).ab. and "review"/)) not (Comment/ or Editorial/ or Letter/ or (animals/ not humans/)) (333116)<br>28 26 and 27 (15)<br>29 Epidemiologic studies/ or case control studies/ or exp cohort studies/ or Controlled Before-After Studies/ or Case control.tw. or (cohort adj (study or studies)).tw. or Cohort analy$.tw. or (Follow up adj (study or studies)).tw. or (observational adj (study or studies)).tw. or Longitudinal.tw. or Retrospective*.tw. or prospective*.tw. or consecutive*.tw. or Cross sectional.tw. or Cross-sectional studies/ or historically controlled study/ or interrupted time series analysis/ or comparative study.pt. (4303867)<br>30 26 and 29 (97)<br>31 exp "Sensitivity and Specificity"/ or (Sensitiv* or Specific*).ti,ab. or (predict* or ROC-curve or receiver-operator*).ti,ab. or (likelihood or LR*).ti,ab. or exp Diagnostic Errors/ or (inter-observer or intra-observer or interobserver or intraobserver or validity or kappa or reliability).ti,ab. or reproducibility.ti,ab. or (test adj2 (re-test or retest)).ti,ab. or "Reproducibility of Results"/ or accuracy.ti,ab. or Diagnosis, Differential/ or Validation Studies.pt. or diagnosis.fs. (7118606)<br>32 26 and 31 (277)<br>33 28 or 30 or 32 (314)	
Embase (Elsevier)	'cerebral blindness'/exp OR 'cerebral visual impairment*':ti,ab OR 'cortical visual impairment*':ti,ab OR 'cognitive visual impairment*':ti,ab OR 'cerebral vision impairment*':ti,ab OR 'cortical vision impairment*':ti,ab OR 'cognitive vision impairment*':ti,ab OR 'cerebral visual disorder*':ti,ab OR 'cortical visual disorder*':ti,ab OR 'cognitive visual disorder*':ti,ab OR (cvi:ti,ab AND (cp:ti,ab OR 'cerebral pals*':ti,ab OR vision:ti,ab OR visual:ti,ab)) OR (brain NEAR/3 ('visual impairment*' OR 'visual disorder*' OR 'vision impairment*')):ti,ab OR 'cortical blindness':ti,ab OR 'cerebral blindness':ti,ab OR ('higher perceptual' NEAR/2 (deficit* OR dysfunction*)):ti,ab<br><br>AND (english)/lim AND (embase)/lim<br><br>AND ('nuclear magnetic resonance imaging'/exp/mj OR (neuroimaging:ti,ab OR 'neuro imaging':ti,ab OR imaging:ti,ab OR mri*:ti,ab OR tractograph*:ti,ab OR hardi:ti,ab OR dwi:ti,ab OR dti:ti,ab OR mr:ti,ab AND imag*:ti,ab) OR neuroradiology:ti,ab OR fmri:ti,ab OR eyetracking:ti,ab)<br>AND ('meta analysis'/de OR cochrane:ab OR embase:ab OR psycinfo:ab OR cinahl:ab OR medline:ab OR (systematic NEAR/1 (review OR overview)):ab,ti OR (meta NEAR/1 analy*):ab,ti OR metaanalys*:ab,ti OR 'data extraction':ab OR cochrane:jt OR 'systematic review'/de) NOT ('animal experiment'/exp OR 'animal model'/exp OR 'nonhuman'/exp NOT 'human'/exp)<br><br>OR 'clinical study'/exp<br><br>OR ('sensitivity and specificity'/de OR sensitiv*:ab,ti OR specific*:ab,ti OR predict*:ab,ti OR 'roc curve':ab,ti OR 'receiver operator':ab,ti OR 'receiver operators':ab,ti OR likelihood:ab,ti OR 'diagnostic error'/exp OR 'diagnostic accuracy'/exp OR 'diagnostic test accuracy study'/exp OR 'inter observer':ab,ti OR 'intra observer':ab,ti OR interobserver:ab,ti OR intraobserver:ab,ti OR validity:ab,ti OR kappa:ab,ti OR reliability:ab,ti OR reproducibility:ab,ti OR (test NEAR/2 're-test'):ab,ti OR (test NEAR/2 'retest'):ab,ti OR 'reproducibility'/exp OR accuracy:ab,ti OR 'differential diagnosis'/exp OR 'validation study'/de OR 'measurement precision'/exp OR 'diagnostic value'/exp OR 'reliability'/exp) (328)	

Figure E Search String Topic five: Genetic assessment

Database	Search terms	Total
Medline (OVID) 2000-juli 2017<br><br>Engels	1 (cerebral visual impairment* or cortical visual impairment* or cognitive visual impairment*).ti,ab,kf. (257)<br>2 (cerebral vision impairment* or cortical vision impairment* or cognitive vision impairment*).ti,ab,kf. (5)<br>3 (cerebral visual disorder* or cortical visual disorder* or cognitive visual disorder*).ti,ab,kf. (8)<br>4 (CVI and (CP or vision or visual)).ti,ab,kf. (151)<br>5 ((brain adj3 ("visual impairment*" or "visual disorder*" or "vision impairment*")) or cortical blindness).ti,ab,kf. (1111)<br>6 Blindness, Cortical/ (539)<br>7 1 or 2 or 3 or 4 or 5 or 6 (1613)<br>10 mutation/ or dna copy number variations/ (386322)<br>11 exp congenital abnormalities/ or genetic diseases, inborn/ (561815)<br>12 exp Metabolic Diseases/ (928979)<br>13 nervous system malformations/ or "agenesis of corpus callosum"/ or central nervous system vascular malformations/ or dandy-walker syndrome/ or hydranencephaly/ or exp "malformations of cortical development"/ or neural tube defects/ or septo-optic dysplasia/ (26487)<br>14 genetics.fs. (3016876)	458

	15 exp Brain Diseases/cn, ge, mi (Congenital, Genetics, Microbiology) (103562)<br>16 Oligonucleotide Array Sequence Analysis/ (64186)<br>17 exp Sequence Analysis, DNA/ (198256)<br>22 (genetic* or heritable or hereditary or chromosome* or mutation* or syndrome* or DNA or "copy number variant" or (metabolic adj (disorder* or disease*)) or (("developmental disorder" or malformation*) adj3 (brain or cortical or "nervous system")) or array* or "exome sequencing" or "agenesis of corpus callosum" or dandy-walker or hydranencephaly or "neural tube" or "septo-optic dysplasiaor" or ((genetic or metabolic) adj (test* or investigation*))).ti,ab,kf. (3082255)<br>23 10 or 11 or 12 or 13 or 14 or 15 or 16 or 17 or 22 (5757642)<br>24 7 and 23 (511)<br>25 limit 24 to (english language and yr="2000 -Current") (300)<br>26 remove duplicates from 25 (289)	
Embase (Elsevier)	'cerebral blindness'/exp OR 'cerebral visual impairment*':ti,ab OR 'cortical visual impairment*':ti,ab OR 'cognitive visual impairment*':ti,ab OR 'cerebral vision impairment*':ti,ab OR 'cortical vision impairment*':ti,ab OR 'cognitive vision impairment*':ti,ab OR 'cerebral visual disorder*':ti,ab OR 'cortical visual disorder*':ti,ab OR 'cognitive visual disorder*':ti,ab OR (cvi:ti,ab AND (cp:ti,ab OR 'cerebral pals*':ti,ab OR vision:ti,ab OR visual:ti,ab)) OR (brain NEAR/3 ('visual impairment*' OR 'visual disorder*' OR 'vision impairment*')):ti,ab OR 'cortical blindness':ti,ab OR 'cerebral blindness':ti,ab OR ('higher perceptual' NEAR/2 (deficit* OR dysfunction*)):ti,ab<br><br>AND (english)/lim AND (embase)/lim<br><br>AND ('gene mutation'/exp/mj OR 'chromosome mutation'/exp/mj OR 'copy number variation'/exp/mj OR 'congenital disorder'/exp/mj OR 'genetic disorder'/exp/mj OR 'inborn error of metabolism'/exp/mj OR 'brain disease'/exp/dm_cn OR 'dna microarray'/exp/mj OR 'dna sequence'/exp/mj OR genetic*:ti,ab OR heritable:ti,ab OR hereditary:ti,ab OR chromosome*:ti,ab OR mutation*:ti,ab OR syndrome*:ti,ab OR dna:ti,ab OR 'copy number variant':ti,ab OR (metabolic:ti,ab AND adj:ti,ab AND (disorder*:ti,ab OR disease*:ti,ab)) OR (('developmental disorder' OR malformation*) NEAR/3 (brain OR cortical OR 'nervous system')):ti,ab OR array*:ti,ab OR 'exome sequencing':ti,ab OR 'agenesis of corpus callosum':ti,ab OR 'dandy walker':ti,ab OR hydranencephaly:ti,ab OR 'neural tube':ti,ab OR 'septo-optic dysplasiaor':ti,ab OR ((genetic OR metabolic) NEAR/1 (test* OR investigation*)):ti,ab) AND (2000-2017)/py<br><br>NOT 'conference abstract':it AND (2000-2017)/py (354)	
